# Efficient Osmotic Energy Conversion Enabled by Self‐Standing COF Membranes With Varied Sulfonic Acid Group Density

**DOI:** 10.1002/adma.73758

**Published:** 2026-06-17

**Authors:** Xi Ma, Xiaoxiao Cheng, Tamara Fischer, Jürgen Senker, Qi Sun, Seema Agarwal

**Affiliations:** ^1^ Advanced Sustainable Polymers Macromolecular Chemistry 2 and Bavarian Polymer Institute University of Bayreuth Bayreuth Germany; ^2^ Zhejiang Provincial Key Laboratory of Advanced Chemical Engineering Manufacture Technology College of Chemical and Biological Engineering Zhejiang University Hangzhou China; ^3^ Department of Chemistry Inorganic Chemistry III, and Northern Bavarian NMR Centre University of Bayreuth Bayreuth Germany

**Keywords:** covalent organic frameworks, ionic membrane, ion separation, osmotic energy

## Abstract

The Gibbs free energy generated from the mixing of seawater and freshwater across a salinity gradient is considered one of the most significant yet underutilized renewable energy sources. Membrane‐based reverse electrodialysis (RED) enables direct electricity generation from osmotic energy by harnessing the net ion flux driven by concentration gradients across ion‐selective membranes. However, entropy generation caused by non‐selective ion mixing significantly limits the power density of RED systems. Therefore, enhancing membrane ion selectivity is critical. 2D covalent organic frameworks (COFs) demonstrate remarkable potential for osmotic energy conversion due to their aligned 1D nanochannel, high porosity, and organized ionic groups. Herein, we present a strategy leveraging electrostatic repulsion to controllably fabricate TpPa‐(SO_3_H)_X_ COF (*X* = 0.5, 1, 1.5, 2) membranes with varied ionic group density. Via stoichiometric modulation during COF synthesis, we achieved variation in sulfonic acid group density within nanochannels, enabling optimized charge‐governed ion selectivity. Under salinity gradients mimicking seawater/freshwater conditions (0.5 m/0.01 m, NaCl), the device delivered an exceptional power output density of 24.53 W m^−2^, representing a 4.9‐fold enhancement over commercial benchmarks (5 W m^−2^). This study presents a novel method and strategy for the design and application of ion‐selective membranes in mass transport and efficient energy conversion.

## Graphical abstract

Self‐standing, strong sulfonated covalent organic framework membranes with varied ionic‐group density are developed for osmotic energy conversion. Optimized charge‐governed nanochannels enable highly selective ion transport, delivering a power density of 24.53 W m^−^^2^ under seawater/freshwater salinity gradients. The work highlights COF membranes as robust platforms for efficient blue‐energy harvesting.

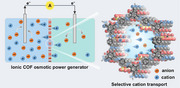

## 1 Introduction

The fossil fuel‐powered Industrial Revolution catalyzed unprecedented technological and economic growth in human history, yet the resultant greenhouse gas emissions and environmental pollution have triggered a severe climate crisis [1, 2]. Against this backdrop, global demand for clean renewable energy is experiencing exponential growth [3]. Osmotic energy, harvested from the salinity gradient between seawater and river water, is regarded as an emerging clean and sustainable energy source [4, 5]. Estimates suggest that the Gibbs free energy released annually from global seawater‐freshwater mixing could theoretically exceed 2.6 TW, equivalent to approximately 17% of global electricity consumption [6, 7]. Compared to solar and wind energy, this abundant “blue energy” is more stable and reliable, exhibits minimal daily fluctuations, and remains unaffected by climatic conditions. Reverse electrodialysis (RED) is the primary technology for capturing this energy, in which ion‐selective membranes serve as the core component of RED systems [8].

Owing to their excellent processability and scalable manufacturing characteristics, polymeric materials play a crucial role in ion‐selective membranes, among which perfluorosulfonic acid (PFSA) polymers represent one of the most representative material systems [9, 10]. PFSA membranes exhibit characteristic microphase‐separated structures, self‐assembled from hydrophilic ion‐conducting domains and hydrophobic polymer matrices [11, 12]. However, the ambiguous interfacial regions between these phases result in the long and discontinuous ionic transport channels. More notably, membrane hydration induces excessive swelling of these conduction channels, significantly compromising their ion selectivity [13]. Recent studies have explored the potential of emerging 2D materials [14, 15, 16, 17], including graphene oxide (GO) [18, 19], boron nitride (BN), molybdenum disulfide (MoS_2_) [20, 21], transition metal carbide and nitride (MXene) [22, 23, 24, 25], and so on, as novel ion‐conductive media. Although precise control of interlayer spacing enables excellent ion selectivity, the highly tortuous transport pathways result in significantly reduced ionic conductivity. Therefore, an ideal ion‐selective membrane should possess: (i) architecturally ordered nanochannels to enable low‐energy‐barrier ion diffusion, (ii) high surface charge density for enhanced ion selectivity, and (iii) excellent stability under operational conditions.

Covalent organic frameworks (COFs) are a class of crystalline porous materials assembled from organic molecular building blocks by dynamic covalent bonding, featuring permanently ordered 1D nanochannels and tunable pore‐wall chemistry [26, 27]. In the 2D COFs, 1D nanopores perpendicular to the direction of the covalently connected 2D structure significantly reduce mass transport resistance [28]. This unique structural advantage enables it suitable for a wide variety of applications ranging from highly efficient separation [29, 30, 31] to the storage and conversion of energy [32, 33, 34, 35]. Besides, compared to the randomly oriented, discontinuous nano‐channels of COF powders, membranes are considered to be more promising morphologies to maximize their structural advantages [36, 37]. Currently, the synthesis of 2D COFs ion‐selective membranes is typically confined to interfacial molecular pre‐assembly strategies to compensate for the substantial entropy loss during monomer polymerization into thin membranes [38, 39, 40]. However, this interface‐confined polymerization approach exhibits self‐terminating characteristics, yielding only fragile ultrathin membranes with thicknesses limited to several tens of nanometers [41, 42]. For practical applications, these nanoscale membranes require substrate support to achieve sufficient mechanical strength. Therefore, the preparation of large‐area continuous and robust self‐standing COF membranes remains a challenge.

In this work, we report an electrostatic repulsion‐mediated synthesis strategy for TpPa‐(SO_3_H)_X_ COF (*X* = 0.5, 1, 1.5, 2) nanosheets, which can be subsequently assembled into defect‐free, robust, and crystalline self‐standing TpPa‐(SO_3_H)_X_ (*X* = 0.5, 1, 1.5, 2) COF membranes via solvent evaporation. Via stoichiometric modulation during COF synthesis, we achieve varied sulfonic acid density in nanochannels, enabling optimized charge‐governed ion selectivity. Under salinity gradients mimicking seawater/freshwater conditions (0.5 m/0.01 m, NaCl), the device delivered an exceptional power output density of 24.53 W m^−2^, representing a 4.9‐fold enhancement over commercial benchmarks (5 W m^−2^).

## 2 Results and Discussion

### 2.1 Synthesis of TpPa‐(SO_3_H)_X_ COF Nanosheets Colloidal Suspension

**FIGURE 1:**
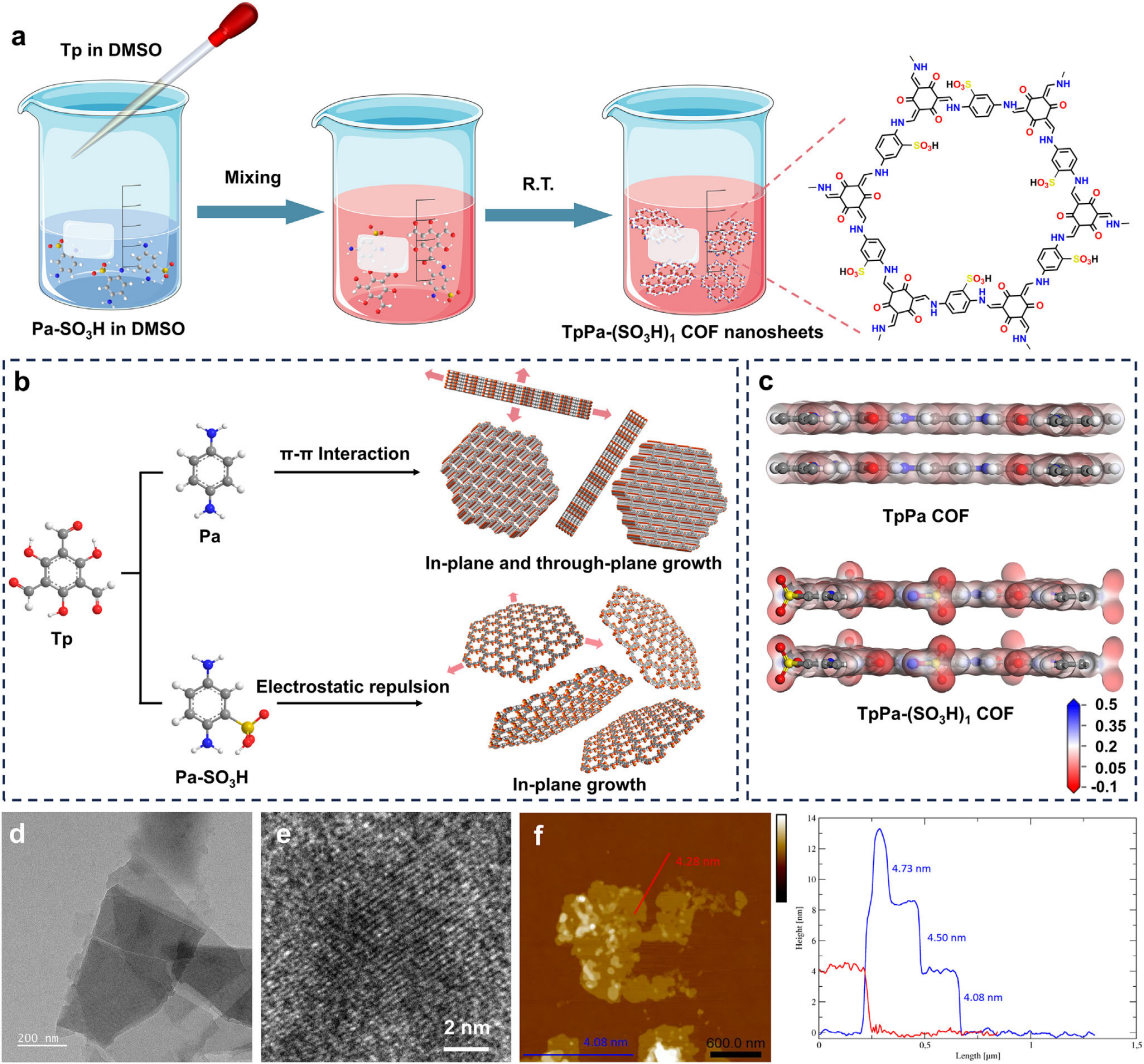
Synthesis and structure of TpPa‐(SO_3_H)_1_ COF nanosheets. (a) Schematic illustration of the TpPa‐(SO_3_H)_1_ COF nanosheets preparation. (b) Schematic representation of the growth of TpPa neutral frameworks and TpPa‐(SO_3_H)_1_ anionic frameworks. TpPa neutral frameworks form aggregates and grow randomly due to π–π interactions, and TpPa‐(SO_3_H)_1_ anionic frameworks lead to in‐plane dominated growth due to electrostatic repulsion resulting in the formation of nanosheets. (c) The distributions of electrostatic potential (ESP) of TpPa and TpPa‐(SO_3_H)_1_. (d) TEM image of TpPa‐(SO_3_H)_1_ COF nanosheets. (e) The high‐resolution TEM image of TpPa‐(SO_3_H)_1_ COF nanosheets. f) AFM image and corresponding height profile of TpPa‐(SO_3_H)_1_ COF nanosheets.

The preparation process for the TpPa‐(SO_3_H)_1_ COF nanosheets is shown exemplarily in Figure 1a and the experimental details are given in Schemes S1–S4. Dimethyl sulfoxide (DMSO) solution dissolving 1,3,5‐triformylphloroglucinol (Tp) monomer was added dropwise to DMSO solution dissolving 2,5‐diaminobenzenesulfonic acid (Pa‐SO_3_H) monomer, and the color of the system turned red immediately after the two monomers were mixed, indicating that the two monomers reacted rapidly. Subsequently, the mixed solution was maintained at room temperature for 24 h to allow the monomers to fully react. The color of the system gradually deepened with the extension of time, and no precipitate was generated in the system (Figure S1a,b). The situation became different when Pa‐SO_3_H was replaced by *p*‐phenylenediamine (Pa). Upon addition of Tp to the Pa solution, the system immediately turned turbid, followed by significant precipitate formation after 24 h (Figure S1c,d). Due to the π–π stacking interactions between neighboring COF monolayers, the newly generated COF crystals tend to spontaneously aggregate to form micrometer‐sized particles [43]. The introduction of sulfonate groups into the framework generates strong interlayer Coulombic repulsion, which effectively suppresses π–π stacking between adjacent COF layers [44]. This electrostatic stabilization promotes preferential in‐plane crystalline growth, yielding crystalline nanosheets with micrometer‐scale lateral dimensions (Figure 1b). Based on the molecular dynamics simulation method, the electrostatic potential distributions of the TpPa COF and TpPa‐(SO_3_H)_1_ COF systems were calculated and comparatively analyzed, and the results obtained effectively verified the previous inference (Figure 1c). Quantitative comparison of electrostatic potential surfaces revealed significant disparities between the two COF systems, with sulfonic acid group‐functionalized regions in the TpPa‐(SO_3_H)_1_ framework demonstrating pronounced charge localization phenomena. The chemical structure evolution and crystal structure characteristics of TpPa particles and TpPa‐(SO_3_H)_1_ nanosheets were verified by Fourier transform infrared spectroscopy (FTIR, Figures S2 and S3) and x‐ray diffraction (XRD, Figure S4) analysis. The experimental data show that the inter‐monomer polycondensation reaction process is complete, and the crystalline COF materials with long‐range ordering have been successfully constructed.

Transmission electron microscopy (TEM) characterization revealed micrometer‐scale sheet‐like morphology of TpPa‐(SO_3_H)_1_ nanosheets (Figure 1d). High‐resolution TEM (HRTEM) imaging identified well‐defined lattice fringes with an interplanar spacing of approximately 0.33 nm (Figure 1e), corresponding to the (001) crystallographic plane, which unambiguously confirmed the highly ordered crystalline structure of the COF nanosheets [45]. The TpPa‐(SO_3_H)_1_ colloidal suspension was deposited on a silicon wafer via drop‐casting method for atomic force microscopy (AFM) characterization. Tapping mode height profile measurements revealed uniformly distributed nanosheet thicknesses centered at 4.5 ± 0.5 nm (Figure 1f). The combination of micrometer‐scale lateral dimensions and nanometer‐level thickness endowed the nanosheets with pronounced structural anisotropy, exhibiting an aspect ratio as high as ∼200. Based on the above studies, four COF nanosheets with varied sulfonic acid group densities (denoted as TpPa‐(SO_3_H)_X_, *X* = 0.5, 1, 1.5, 2) were successfully fabricated through a stoichiometrically controlled functionalization strategy. It is worth noting that when preparing TpPa‐(SO_3_H)_1.5_ and TpPa‐(SO_3_H)_2_ COF nanosheets, the temperature needs to be raised from room temperature to 120°C due to the weak reactivity of Pa‐(SO_3_H)_2_. All the four COF colloidal suspensions prepared showed significant Tyndall effect indicating the formation of nanosheets (Figure S5). The zeta potential of Pa‐(SO_3_H)_X_ COF nanosheets became more negative with increasing sulfonic acid group density (Figure S6).

### 2.2 Preparation and Characterization of TpPa‐(SO_3_H)_X_ COF Membranes

**FIGURE 2:**
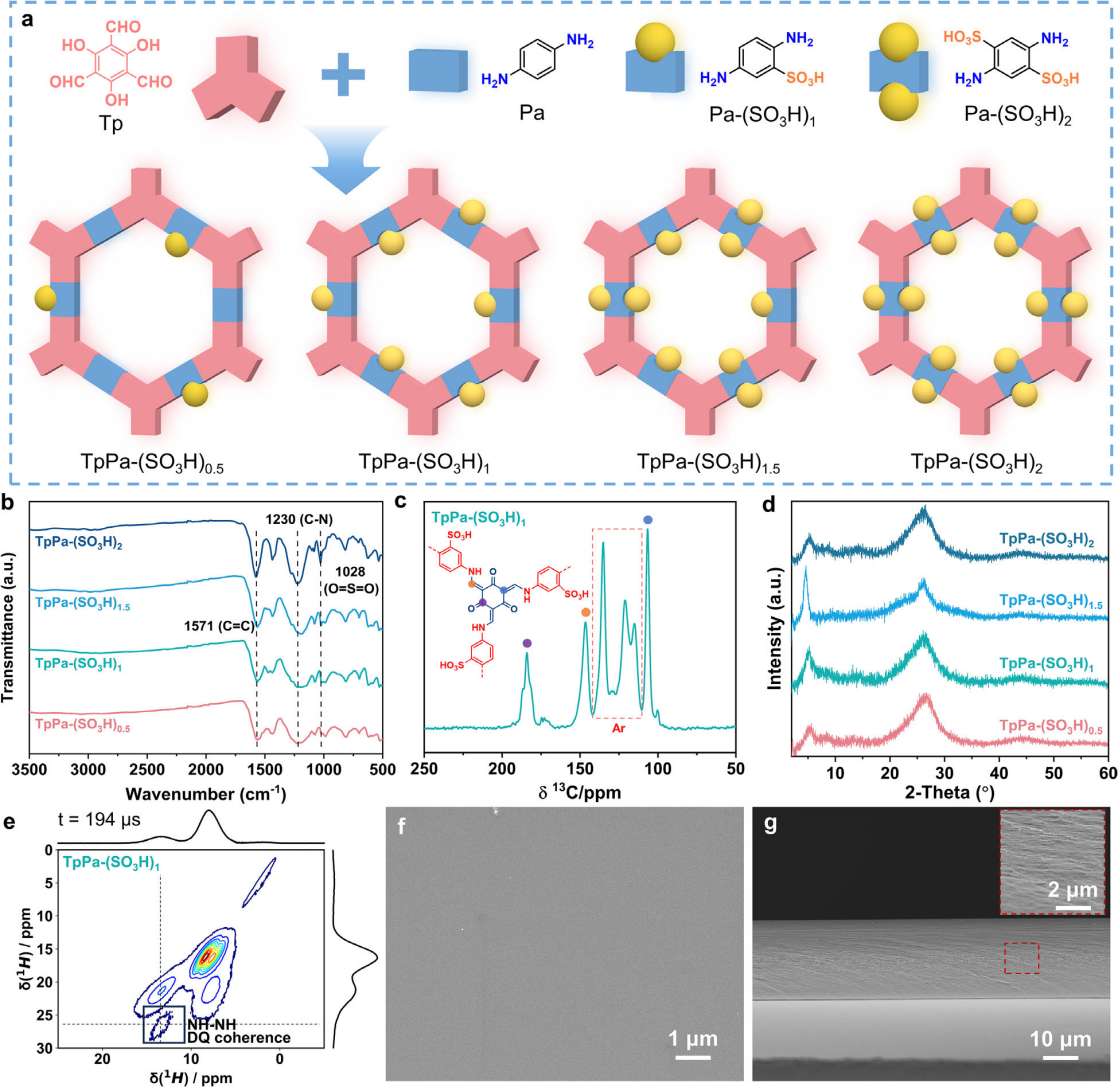
Structural and morphology characterizations of the TpPa‐(SO_3_H)_X_ COF membranes. (a) The structure of four COF membranes in this work. (b) FT‐IR spectra of the TpPa‐(SO_3_H)_X_ COF membranes. (c) ^13^C solid‐state NMR spectra of the TpPa‐(SO_3_H)_1_ COF membrane. (d) PXRD patterns of the TpPa‐(SO_3_H)_X_ COF membranes. (e) ^1^H‐^1^H DQ‐SQ spectrum of TpPa‐(SO_3_H)_1_ COF membrane with a recoupling‐time of 194 µs and highlighted NH‐NH DQ coherence. (f) Top‐view and (g) cross‐sectional view SEM images of the TpPa‐(SO_3_H)_1_ COF membrane. Inset is a higher magnification image for the dash‐line area.

Self‐standing COF membranes can be easily obtained by solvent evaporation of COF nanosheets suspension (Figure S7). The as‐prepared COF membranes exhibited remarkable structural integrity and flexibility, mechanical tests revealed outstanding tensile strength (65–89 MPa) for TpPa‐(SO_3_H)_X_ COF membranes, surpassing most polymer‐based ion‐selective membranes as well as state‐of‐the‐art self‐standing COF membranes (Table S1 and Figure S8). Furthermore, no structural failure was observed after folding, rolling, and twisting treatments (Figure S9). All scanning electron microscopy (SEM) characterization revealed that the surface morphology of the COF membrane was intact and free of macroscopic cracks and microscopic pinhole defects. Cross‐sectional analysis further demonstrated a densely homogeneous architecture with a membrane thickness of approximately 20 µm (Figure 2f,g and Figures S10–S12). As the COF nanosheets are assembled through a solvent‐evaporation‐induced process, they can gradually and uniformly organize into a continuous membrane, minimizing defects and promoting the formation of a dense structure. This process is distinct from the conventional assembly of 2D material‐based membranes, such as MXene, MoS_2_, or GO membranes, which often involves rapid deposition, for example by vacuum filtration, and can trap voids or lead to less ordered stacking. Systematic characterization by FT‐IR and ^13^C solid‐state nuclear magnetic resonance (NMR) analyses confirmed that all membrane samples exhibited COF characteristic β‐ketoenamine bond absorption bands (Figure 2b and Figures S13 and S14) and chemical shift distributions (Figure 2c, Figure S15) [46, 47]. Furthermore, the stretching vibration band assigned to the O═S═O bond at 1028 cm^−1^ was observed in all TpPa‐(SO_3_H)_X_ COF membranes [48], and its intensity increased progressively with the feed ratio of the Pa‐(SO_3_H)_2_ monomer. This confirms the successful incorporation of sulfonic acid groups with varying contents in these COFs. Furthermore, the sulfur contents determined by elemental analysis show a systematic increase from ∼6.5 to ∼10 wt.% for the TpPa‐(SO_3_H)_X_ membranes (Table S2). The experimentally obtained values are lower than the theoretical values, particularly for membranes containing highly sulfonated monomer Pa‐(SO_3_H)_2_. This deviation is likely attributable to the increased steric hindrance associated with multiple sulfonic acid groups, which reduces the effective incorporation efficiency of this monomer. A similar trend is observed for COF powder samples synthesized by the solvothermal method, indicating that this behavior originates from the intrinsic reactivity of the monomers rather than from the specific membrane‐fabrication method. In the powder x‐ray diffraction (PXRD) patterns of the TpPa‐(SO_3_H)_X_ COF membranes, the occurrence of the (100) and (001) reflections at 4.7° and 26.0° matches literature‐reported values, suggesting a layered structure composed of planar hexagonal COF sheets (Figure 2d) [49]. To probe whether adjacent layers are still stacked in an AA‐type fashion, a suite of ^1^H‐^1^H double‐quantum (DQ) single‐quantum (SQ) spectra was recorded with recoupling times between 48 and 292 µs (Figures S16–S19). In this regime, only the strongest dipolar couplings and thus the shortest H‐H distances are recorded. Exemplarily, one DQ spectrum for TpPa‐(SO_3_H)_1_ with *t*_r_ = 194 µs is displayed in Figure 2e. The spectra are dominated by CH‐CH (15.9 ppm) and CH‐NH (21.3 ppm) DQ coherences with short distances within one layer. In addition, a DQ coherence at 26.8 ppm was observed, corresponding to NH‐NH couplings that can only occur between adjacent layers for the chosen recoupling times. This DQ coherence was already observed for the shortest recoupling time of 48 µs, suggesting that for the majority of layers at least one adjacent layer with a distance around 3.5 Å is present and that these layers are stacked in an AA‐typed fashion [50]. Water contact angle measurements revealed that as the sulfonic acid group content increased, the water contact angle of the TpPa‐(SO_3_H)_X_ COF membranes decreased from 69° to 47° (Figure S20). This enhanced wettability thereby effectively promoted ion and water transport. Water stability of the ion‐selective membrane is crucial for efficient osmotic energy harvesting. Remarkably, even the TpPa‐(SO_3_H)_2_ COF membrane with the highest sulfonic acid group density maintained its structural integrity and showed no detectable change in dimensions after 30 days of immersion in deionized water (Figure S21).

### 2.3 Investigation on the Ion Transmembrane Behavior of TpPa‐(SO_3_H)_X_ COF Membranes

**FIGURE 3:**
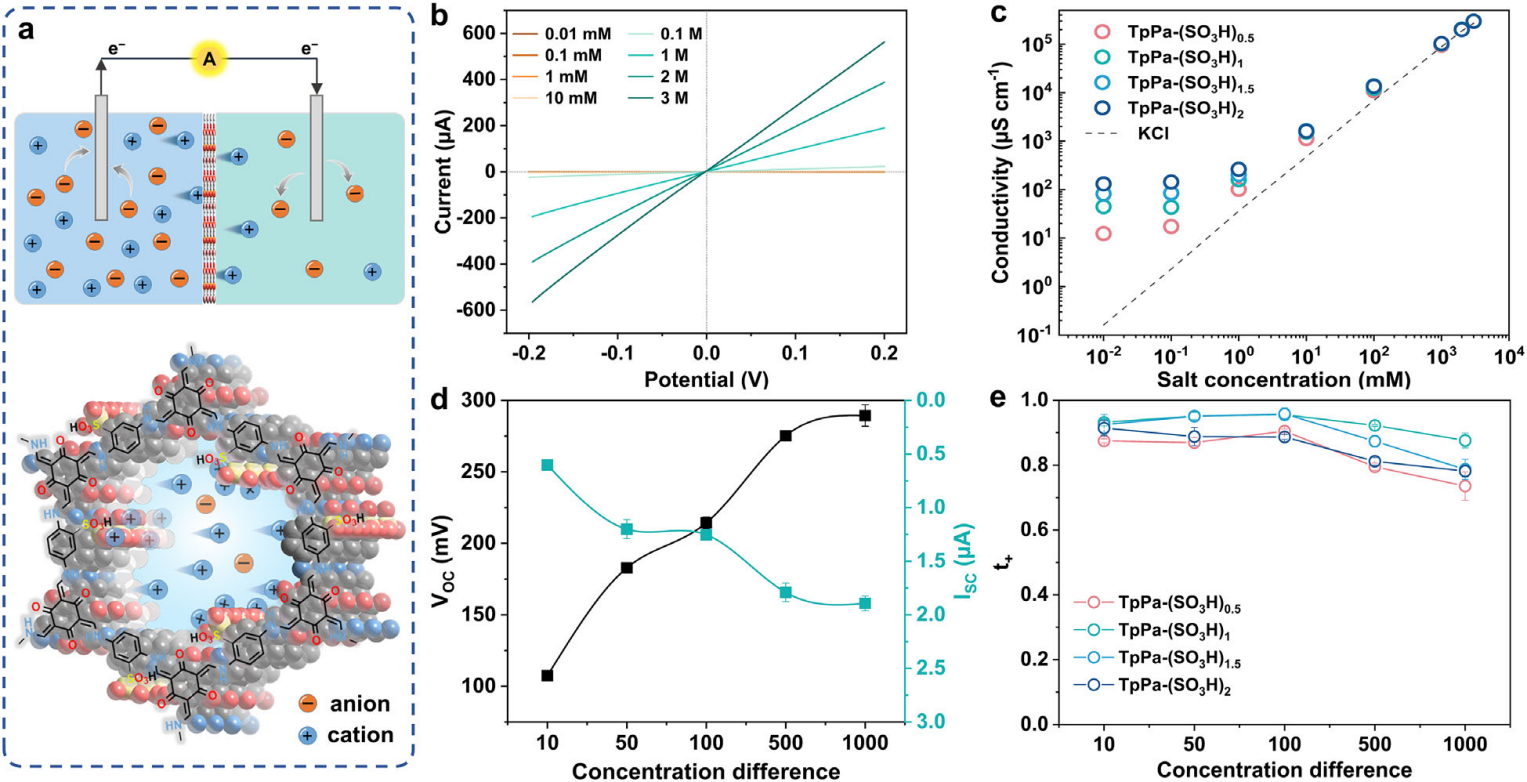
Investigation of ion transmembrane behavior. (a) Schematic of setup used for the investigation of ion transport behavior across the fabricated membranes. (b) *I–V* curves for TpPa‐(SO_3_H)_1_ recorded using KCl solutions with concentrations ranging from 0.01 mm to 3 m. (c) Ion conductivity of TpPa‐(SO_3_H)_1_ as a function of electrolyte (KCl) concentration. (d) Plots of the recorded *V*_OC_ and *I*_SC_ versus differences in KCl concentration across TpPa‐(SO_3_H)_1_, and (e) plots of transference number (*t*_+_) versus differences in KCl concentration across TpPa‐(SO_3_H)_x_.

To evaluate the ion permeability and selectivity of the TpPa‐(SO_3_H)_X_ COF membranes, ion transport behavior across the membrane was investigated. As shown in Figure 3a, KCl solutions of the same concentration were separated by a COF membrane and connected to an external circuit using a pair of homemade Ag/AgCl electrodes. The ionic current at different salinities was then recorded using linear sweep voltammetry. Here, KCl is used as the electrolyte solution because its cation (K^+^, 1.960 × 10^−9^ m^2^ s^−1^) and anion (Cl^−^, 2.032 × 10^−9^ m^2^ s^−1^) have similar ion mobility. Figure 3b and Figure S22 display current‐voltage (*I*–*V*) curves measured using KCl solutions with concentrations ranging from 0.01 mm to 3 m. All membranes exhibited the characteristic linear ohmic response of symmetric nanofluidic membranes, indicative of their symmetric microstructure. The correlation between ionic conductivity and electrolyte concentration reveals the ion transport behavior controlled by surface charge (Figure 3c). Specifically, at high electrolyte concentrations, the excess ions near the membrane surface screen the surface charge effectively. Consequently, the ionic conductivity follows the bulk behavior, increasing linearly with electrolyte concentration. However, when the concentration falls below 0.1 m, the Debye length increases. Under these dilute conditions, the ion concentration within the channels is governed by the material's surface charge density, causing the ionic conductivity to deviate from the bulk value and saturation. Notably, this deviation becomes more pronounced with increasing sulfonic acid group density.

The ion selectivity of COF membranes was quantitatively evaluated using the ion transfer number. The TpPa‐(SO_3_H)_X_ membrane was mounted between two diffusion cells filled with KCl solutions of different concentrations. One reservoir was fixed at 0.1 mm KCl, while the concentration in the opposing reservoir was systematically varied from 1 mm to 0.1 m KCl. For each established concentration gradient, the *I*–*V* curve across the membrane was measured. The open‐circuit (*V*_OC_) voltage and short‐circuit current (*I*_SC_) can be obtained directly from the *I*–*V* curves by noting the values at zero current for *V*_OC_ and zero voltage for *I*_SC_ (Figure S23). Figure 3d and Figure S24 record the *V*_OC_ and *I*_SC_ under different KCl concentration gradients. The results show that the *V*_OC_ and *I*_SC_ of all COF membranes increased with the KCl concentration difference. Notably, the TpPa‐(SO_3_H)_1_ membrane consistently exhibited higher *V*_OC_ and *I*_SC_ than the other membranes across all tested concentration differences. The *V*_OC_ arises from the flux difference between anions and cations during selective transmembrane transport. Based on the Nernst equation, the cation transference number (*t*_+_) was calculated to quantitatively evaluate the ion selectivity of the four membranes (Table S3 and Figure S25). As shown in Figure 3e, the cation transference number of the ion‐selective membranes with different ionic group densities decreases with increasing concentration gradient. This decrease is primarily attributed to the compression of the electrical double layer at higher electrolyte concentrations, which increases the resistance to ion transmembrane transport. Among them, the TpPa‐(SO_3_H)_1_ membrane exhibited the highest *t*_+_, maintaining a value of 0.922 even under an ultrahigh concentration gradient of 500‐fold. This exceptional ion selectivity is attributed to its negatively charged nanochannels, which facilitate the permeation of hydrated potassium cations (K^+^) through electrostatic attraction while effectively repelling chloride anions (Cl^−^) via electrostatic repulsion. Such highly selective ion transport mitigates the entropy increase associated with uncontrolled mixing of high‐concentration saline solutions, paving the way for broader application prospects.

### 2.4 Performance Evaluation of the Osmotic Energy Conversion of TpPa‐(SO_3_H)_X_ COF Membranes

**FIGURE 4:**
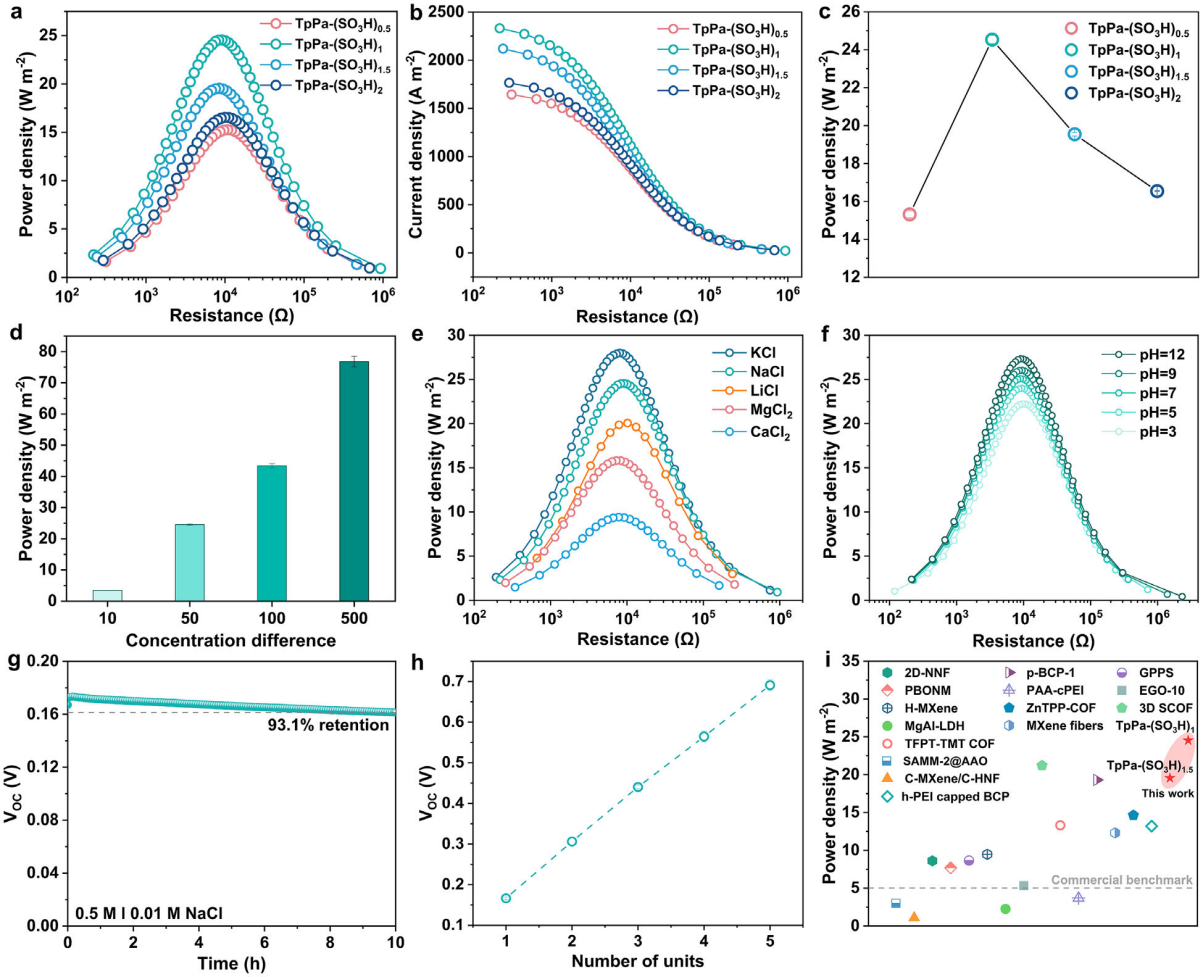
Osmotic energy conversion performance. (a) The output power density and (b) current density of the TpPa‐(SO_3_H)_X_ membranes as a function of the external load resistance under a salinity gradient of 0.5 m/0.01 m NaCl. c) The maximum output power density of RED system based on TpPa‐(SO_3_H)_X_ membranes. (d) The maximum output power density of RED system based on TpPa‐(SO_3_H)_1_ membrane at different concentration folds. The low‐salinity NaCl solution is fixed at 0.01 m, and high‐salinity is varied from 0.1 to 5 m. (e) The output power density at different electrolytes of the TpPa‐(SO_3_H)_1_ membrane as a function of the external load resistance under a 50‐fold salinity gradient (0.5 m/0.01 m). (f) The output power density at different pH values of the TpPa‐(SO_3_H)_1_ membrane as a function of the external load resistance under a salinity gradient of 0.5 m/0.01 m NaCl. (g) *V*_OC_‐*t* curve of the TpPa‐(SO_3_H)_1_‐RED system under a salinity gradient of 0.5 m/0.01 m NaCl. After 10 h of continuous operation without electrolyte replenishment, the performance retention rate was 93.1%. (h) The linear relationship between voltage and the different units of the TpPa‐(SO_3_H)_1_‐RED system. (i) The osmotic energy conversion performance of TpPa‐(SO_3_H)_1_ membrane and TpPa‐(SO_3_H)_X_ membrane compared with the most advanced osmotic generator.

Motivated by the excellent ion selectivity of the TpPa‐(SO_3_H)_X_ membrane, we proceeded to systematically evaluate its osmotic energy conversion capability. A chemical potential gradient was established in the RED system using 0.5 m (simulated seawater) and 0.01 m (simulated freshwater) NaCl solutions. The relationship between power density and external resistance is shown in Figure 4a, with the power density reaching its peak when the internal resistance of the membrane was equal to the external load resistance. Among them, the TpPa‐(SO_3_H)_1_ membrane showed the highest power density output of 24.53 W m^−2^. Both TpPa‐(SO_3_H)_1_ and TpPa‐(SO_3_H)_1.5_ exhibit similar membrane resistance, which is substantially lower than that of TpPa‐(SO_3_H)_0.5_ and TpPa‐(SO_3_H)_2_. This low‐resistance characteristic contributes to enhanced osmotic energy harvesting. The osmotic energy conversion performance can be modulated by adjusting the ionic group density of the ion‐selective membranes. All membranes showed similar trends, as shown in Figure 4b, with the current density decreasing as the external load resistance increased. As shown in Figure 4c, the power density exhibits a trend of first increasing and then decreasing as a function of ionic group density. The COF membrane with an intermediate ionic group density demonstrates optimal osmotic energy conversion performance. This counterintuitive surface‐charge‐dependent osmotic power behavior is also observed in other osmotic energy conversion systems with high ionic group density [51, 52, 53]. The low osmotic power generated by nanopores with high surface charge primarily stems from pronounced concentration polarization effects, characterized by ion depletion at the high‐concentration entrance and ion enrichment at the low‐concentration exit. Furthermore, concentration polarization intensifies with increasing surface charge density, resulting in a significant reduction of the effective salinity gradient.

As evidenced by the results above, the TpPa‐(SO_3_H)_1_ membrane exhibited optimal osmotic energy conversion performance and was thus selected for subsequent experimental investigations. Subsequently, the osmotic energy conversion performance of the TpPa‐(SO_3_H)_1_ membrane‐based RED system under different salinity gradients was investigated by fixing the NaCl concentration on the low‐salinity side at 0.01 m and gradually increasing the concentration on the high‐salinity side from 0.1 to 5 m. Both the output power density and current density of the system increased continuously with the growing salinity gradient, reaching a power density of 76.8 W m^−2^ at a 500‐fold gradient (Figure 4d, Figure S26). The increase in the salinity gradient enhances the driving force for ion transport across the membrane, resulting in greater power output. These results indicate that our TpPa‐(SO_3_H)_1_ ion‐selective membrane exhibits promising potential for applications across a wide range of salt concentrations. Given that the energy conversion behavior in RED systems is also correlated with the ionic diffusion coefficients of electrolytes, this study systematically investigates the osmotic energy conversion characteristics of TpPa‐(SO_3_H)_1_‐based RED systems with different electrolytes under identical salinity gradients (0.5 m/0.01 m). As depicted in Figure 4e and Figure S27, the output power density and current density of the TpPa‐(SO_3_H)_1_ membrane under different electrolytes exhibit consistent variation trends with external load resistance. The maximum power densities for the four electrolytes are 28.4 W m^−2^ (KCl), 24.5 W m^−2^ (NaCl), 19.7 W m^−2^ (LiCl), 16.0 W m^−2^ (CaCl_2_) and 9.4 W m^−2^ (MgCl_2_), respectively. All electrolyte systems share identical anions, with their osmotic energy conversion performance primarily governed by cation characteristics. The maximum power output sequence exhibits a positive correlation with the diffusion coefficients of corresponding cations (Table S4). Furthermore, for divalent cations, their higher intrinsic charge induces stronger membrane‐ion electrostatic interactions, which hinder ionic transport and critically limit power generation. The osmotic energy conversion behavior in RED systems is controlled by the surface charge of the ion channels. Given that the TpPa‐(SO_3_H)_1_ ion‐selective channels contain abundant sulfonic acid groups, whose charge characteristics are pH‐dependent, the osmotic energy conversion performance was investigated across different pH values. The pH of the electrolyte solutions was adjusted using dilute HCl and NaOH solutions. Figure 4f and Figure S28 show the variation of output power density and current density of the TpPa‐(SO_3_H)_1_ membrane‐based RED system at different pH values as a function of external load resistance under a salinity gradient of 0.5 m/0.01 m NaCl. The maximum output power density of the system increased with rising pH values and reached its peak value of 28.0 W m^−2^ at pH 12. This trend is consistent with an enhanced negative charge environment in the nanochannels at higher pH, which may promote cation‐selective transport. However, because pH adjustment with HCl and NaOH simultaneously changes the ionic composition and counterion environment, the observed performance variation should be regarded as an apparent pH‐dependent effect rather than a purely isolated change in fixed charge density.

Long‐term stability is crucial for practical applications. To evaluate this, we conducted a long‐term stability test on the TpPa‐(SO_3_H)_1_ ion‐exchange membrane by continuously recording the *V*_OC_ of the RED system (0.5 m NaCl/0.01 m NaCl) for 10 h without replenishing the electrolytes. The performance remained stable throughout the test, with a high *V*_OC_ retention rate of 93.1% (Figure 4g). Meanwhile, the well‐preserved crystalline structure of the COF membrane maintains the integrity of the ion transport pathways, enabling stable long‐term osmotic energy conversion (Figure S29). These results demonstrate the excellent operational stability of the COF ion‐selective membrane. To validate the scalability of the RED system, we constructed a RED stack comprising five unit cells connected in series to achieve higher output voltage (Figure S30). As shown in Figure 4h, the output voltage exhibited a strong linear relationship with the number of series‐connected units, reaching 0.706 V for the five‐unit stack. This result demonstrates the excellent application potential of our COF membrane‐based osmotic energy conversion system. The TpPa‐(SO_3_H)_X_ membrane achieves a remarkable power density, outperforming many advanced systems documented in the literature (Figure 4i and Table S5). This highlights the great potential of COF materials with ordered ion channels and robust frameworks for future high‐performance osmotic energy conversion applications.

## 3 Conclusion

In summary, we developed a robust, crystalline, and self‐standing COF membrane fabrication strategy featuring a variation in SO_3_H group density. We systematically investigated how the density of these groups within the nanochannels influences osmotic energy harvesting performance. By precisely tuning the ionic group density, we found that COF membranes with a moderate density exhibited the highest energy conversion efficiency, achieving a maximum power density of 24.53 W m^−2^ under a 0.5 m NaCl/0.01 m NaCl concentration gradient‐approximately 4.9 times higher than the commercial benchmark. It should be noted that the observed performance trend likely arises from a combination of chemical functionality and structural characteristics, rather than a single independent variable. Moreover, the membrane demonstrated excellent mechanical strength and long‐term stability, meeting the practical requirements for real‐world applications. Beyond osmotic energy conversion, our findings also offer new insights into other membrane‐based energy conversion technologies driven by chemical potential gradients, such as lithium extraction from brines and seawater desalination.

We believe that the electrostatic‐repulsion‐mediated synthesis strategy developed in this work, in principle, extendable to other COF membranes incorporating different ionic functional groups, provided that the charged or ionizable moieties generate sufficient electrostatic interactions to suppress uncontrolled aggregation and promote well‐dispersed nanosheet formation. More generally, the reticular design of COFs enables precise control over pore size, nanochannel chemistry, and functional group distribution, allowing selective ion transport to be regulated through well‐defined molecule–channel interactions rather than through the less‐defined interlayer galleries in conventional 2D nanosheet membranes such as MXene, MoS_2_, and GO. These features make COF membranes a highly versatile platform for osmotic energy conversion and other membrane‐based separation and energy technologies.

## Conflicts of Interest

The authors declare no conflicts of interest.

## Supporting information




**Supporting File**: adma73758‐sup‐0001‐SuppMat.docx.

## Data Availability

The data that supports the findings of this study are available in the supplementary material of this article.
