# Supplementary material for: Efficient Osmotic Energy Conversion Enabled by Self‐Standing COF Membranes With Varied Sulfonic Acid Group Density
Source: Adv Mater. 2026 Jun 17;38(41):e73758. doi: 10.1002/adma.73758 (PMC13393992; doi:10.1002/adma.73758)
Supplement: Supplementary file 1 — Supporting File: adma73758‐sup‐0001‐SuppMat.docx. [file ADMA-38-e73758-s001.docx]

**Supplementary Information**

**Efficient Osmotic Energy Conversion Enabled by Self-Standing COF Membranes with Varied Sulfonic Acid Group Density**

*Xi Ma^1^***, Xiaoxiao Cheng^2^, Tamara Fischer^3^, Jürgen Senker^3^, Qi Sun^2^, Seema Agarwal^1^**

^1^ Advanced Sustainable Polymers, Macromolecular Chemistry 2 and Bavarian Polymer Institute, University of Bayreuth, 95440 Bayreuth, Germany

^2^ Zhejiang Provincial Key Laboratory of Advanced Chemical Engineering Manufacture Technology, College of Chemical and Biological Engineering, Zhejiang University, 310027 Hangzhou, China

^3^ Department of Chemistry, Inorganic Chemistry III, and Northern Bavarian NMR Centre, University of Bayreuth, 95440 Bayreuth, Germany

E-mail: [agarwal@uni-bayreuth.de](mailto:agarwal@uni-bayreuth.de); [xi.ma@uni-bayreuth.de](mailto:xi.ma@uni-bayreuth.de)

**1. Experimental Procedure**

**Materials**

1,3,5-triformylphloroglucinol (Tp, 97%), *p*-phenylenediamine (Pa, 98%), 2,5-diaminobenzenesulfonic acid (Pa-SO_3_H, 98%) and 2,5-diaminobenzene-1,4-disulfonic acid (Pa-(SO_3_H)_2_, 97%) were purchased from BLD Pharm Ltd. Lithium chloride (LiCl, 98%), sodium chloride (NaCl), potassium chloride (KCl), magnesium chloride (MgCl_2_) and calcium chloride (CaCl_2_) were purchased from Sigma-Aldrich. Dimethylsulfoxide (DMSO), tetrahydrofuran (THF), acetone and ethanol were purchased from Fisher Chemical. Ultrapure water was used in all experiments and was supplied by Milli-Q Advantage A10 (Millipore, USA). All reagents were used as received from commercial sources without further purification.

**Characterizations**

The chemical structures of the samples were analyzed with a total reflectance-Fourier transform infrared spectrometer (ATR-FTIR, 400-4000 cm^-1^, Digilab Excalibur FTS-3000). Powder X-ray diffraction (PXRD) patterns were collected on a Panalytical EMPYREAM instrument with Cu Kα radiation (1.54 Å) using a flat-plate Si sample spinner. Each sample was scanned at a rate of 2° min^-1^ in the range of 2θ = 2° to 60°. ^13^C solid-state NMR spectra were acquired on a Bruker Avance III HD spectrometer operating at a B_0_ field of 9.4 T (ν_0_(^13^C) = 100.6 MHz). The samples were spun at 15.0 kHz (^13^C) in a 3.2 mm MAS triple resonance probe. ^13^C MAS spectra were obtained with ramped cross-polarization (CP) experiments where the ^13^C nutation was set to 50 kHz and the ^1^H nutation frequency ν_nut_ was varied linearly from 50‑100% to match the Hartmann‑Hahn conditions. The contact time was set to 3 ms. Proton broadband decoupling with spinal-64 and ν_nut_ = 70 kHz was applied during acquisition. ^1^H-^1^H DQ-SQ experiments were performed using a Bruker Avance III HD spectrometer operating at a magnetic field strength of 14.7 T, corresponding to a ^1^H Larmor frequency of 600.1 MHz. All experiments were performed using a Bruker 1.3 mm double resonance probe at a MAS frequency of 62.5 kHz. The 2D DQ-SQ spectra were recorded using the DQ recoupling sequence ${R12}_{2}^{5}$ with a nutation frequency of 190 kHz for the R sequence and of 150 kHz for the 90° pulse. All spectra are referenced with respect to tetramethylsilane. The absence of any higher-order reflections indicates moderate crystallinity with lateral disorder between individual layers. This disorder is also evident by broadening of the 13C NMR signals at around 120 ppm, which corresponds to the partially sulfonated aromatic carbons. To probe whether adjacent layers are still stacked in an AA-type fashion, a suite of 1H-1H double-quantum (DQ) single-quantum (SQ) spectra was recorded with recoupling times between 48 µs and 292 µs (Figure S15-S18, Supporting Information). In this regime, only the strongest dipolar couplings and thus the shortest H-H distances are recorded. Exemplarily, one DQ spectrum for TpPa-(SO3H)1 with tr = 194 µs is displayed in Figure 2e. The spectra are dominated by CH-CH (15.9 ppm) and CH-NH (21.3 ppm) DQ coherences with short distances within one layer. In addition, a DQ coherence at 26.8 ppm was observed, corresponding to NH-NH couplings that can only occur between adjacent layers for the chosen recoupling times. This DQ coherence was already observed for the shortest recoupling time of 48 µs, suggesting that for the majority of layers at least one adjacent layer with a distance around 3.5 Å is present and that these layers are stacked in an AA-typed fashion.

Scanning electron microscope (SEM, FEI Quanta FEG 250) was used to capture the microscopic morphologies of all obtained membranes. These samples to be observed in cross-section were obtained by brittle fracture in liquid nitrogen, and they need to be sprayed with gold by vacuum sputtering before observation to increase electrical conductivity. High-resolution transmission electron microscopy (HR-TEM) images were obtained on a JEOL 2100F. Atomic force microscopy (AFM, Bruker Dimension Icon) used a tapping mode to obtain information such as the thickness and transversal dimensions of the sample. The TpPa-(SO_3_H)_x_ COF nanosheet suspension was diluted with 0.001 M KCl solution to obtain a stable dispersion (~ 0.1 mg/mL), and its zeta potential was measured using a zeta potential analyzer (Nano ZS90, Malvern, UK). The contact angle goniometer (KRUSS GMBH, Hamburg 100) was used to measure the sessile drop contact angle of water on the membrane samples. The stress-strain curves of the prepared membranes were tested by a universal tensile testing machine (Zwick/Roell, Germany) to evaluate their mechanical properties at a deformation rate of 5 mm min^-1^ at room temperature.

**Electrostatic potential calculation**

The electrostatic potential of TpPa COF and TpPa-(SO_3_H)_1_ COF was calculated using the DMol^3^ module in Materials Studio 2023 software. The exchange-correlation energy was calculated using the Perdew-Burke-Ernzerhof (PBE) generalized gradient approximation (GGA) functional in electronic structure simulations.^[1]^

**Synthesis of TpPa COF particles**

Pa (0.15 mmol, 16.22 mg) and Tp (0.1 mmol, 21.01 mg) were dissolved separately, each in 2.5 mL DMSO to obtain aldehyde and amine solution, respectively. After dissolution, the aldehyde solution was added dropwise into the amine solution under gentle stirring, immediately resulting in abundant precipitate formation within the system. Then, this system was maintained stationary at room temperature for 24 h. Subsequently, the precipitate was collected via centrifugation and sequentially washed with DMSO and acetone. The obtained particles were vacuum-dried at 120 °C for 24 h.

**Fabrication of free-standing COF membranes**

COF membranes with different charge densities were synthesized by selecting different molecular building blocks. COF membranes are denoted as COF-(SO_3_H)_x_, where x refers to the initial molar ratio of sulfonic acid group to the diamine compounds.

*1. Fabrication of self-standing TpPa-(SO_3_H)_0.5_ COF membranes*


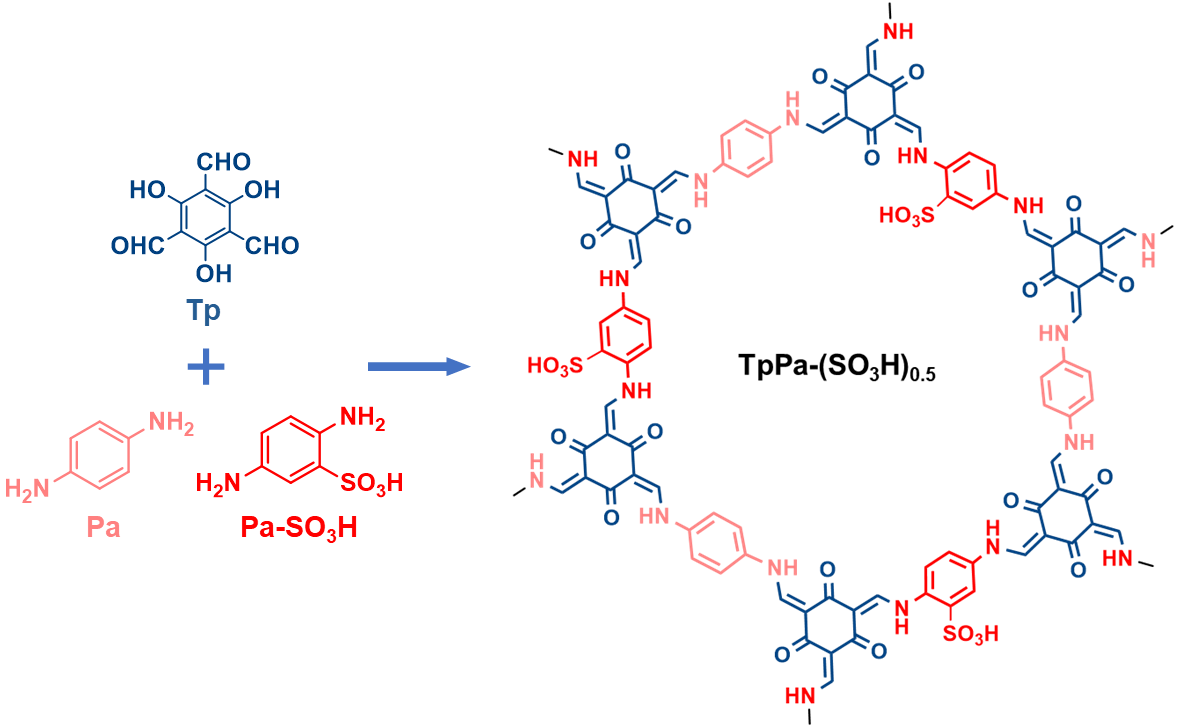


**Scheme S1.** Preparation procedure for TpPa-(SO_3_H)_0.5_.

TpPa-(SO_3_H)_0.5_ preparation scheme is shown in Scheme S1. Pa (0.075 mmol, 8.11 mg) and Pa-SO_3_H (0.075 mmol, 14.11 mg) were dissolved separately, each in 3.5 mL DMSO to obtain amine solution, respectively. Tp (0.1 mmol, 21.01 mg) was dissolved in 7 mL of DMSO to obtain an aldehyde solution. After dissolution, the aldehyde solution was first added dropwise into the Pa-SO_3_H solution, and then the Pa solution is added to the mixed system under gentle stirring. Then, this system was maintained stationary at room temperature for 24 h. Subsequently, the resulting mixed solution was poured into a 60 mm diameter Petri dish and was dried at 80 °C for about 3 days to obtain the self-standing TpPa-(SO_3_H)_0.5_ COF membrane.

*2. Fabrication of self-standing TpPa-(SO_3_H)_1_ COF membranes*


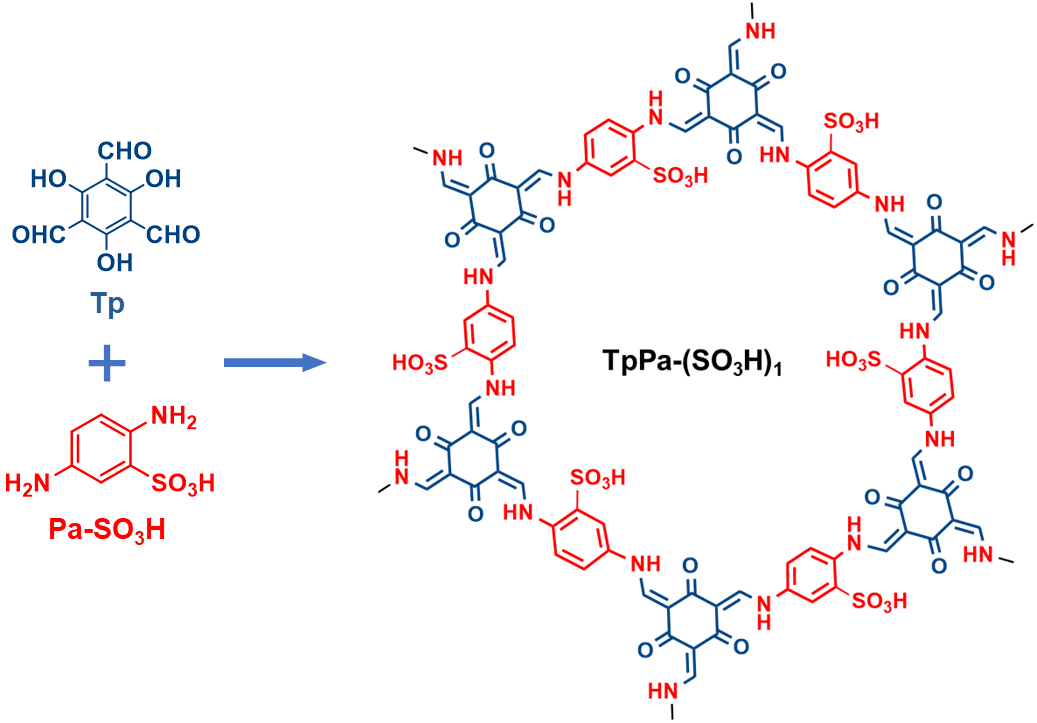


**Scheme S2.** Preparation procedure for TpPa-(SO_3_H)_1_.

Pa-SO_3_H (0.15 mmol, 28.23 mg) and Tp (0.1 mmol, 21.01 mg) were dissolved separately, each in 2.5 mL DMSO to obtain aldehyde and amine solution, respectively. After dissolution, the aldehyde solution was added dropwise into the amine solution under gentle stirring. Then, this system was maintained stationary at room temperature for 24 h, during which time the color of the solution gradually darkened. Subsequently, the resulting mixed solution was poured into a 60 mm diameter Petri dish and was dried at 80 °C for about 3 days to obtain the self-standing TpPa-(SO_3_H)_1_ COF membrane.

*3. Fabrication of self-standing TpPa-(SO_3_H)_1.5_ COF membranes*


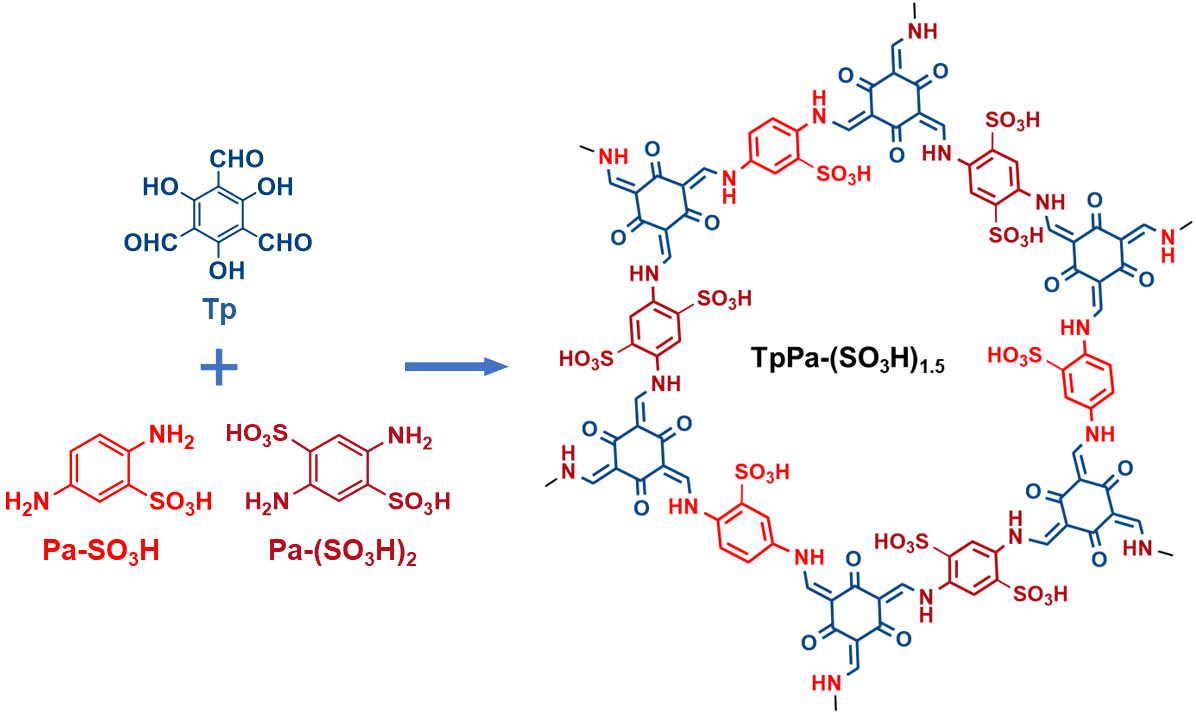


**Scheme S3.** Preparation procedure for TpPa-(SO_3_H)_1.5_.

The Pa-SO_3_H (0.075 mmol, 14.11 mg) and Pa-(SO_3_H)_2_ (0.075 mmol, 20.12 mg) mixture was dissolved in 5 mL of DMSO to obtain an amine solution. Tp (0.1 mmol, 21.01 mg) was dissolved in 5 mL of DMSO to obtain an aldehyde solution. After dissolution, the aldehyde solution was added dropwise into the amine solution under gentle stirring. Then, the mixture was frozen in liquid N_2_, followed by degassing treatment by three freeze-pump-thaw cycles and heated at 120 °C for 3 days. Subsequently, the resulting mixed solution was poured into a 60 mm diameter Petri dish and was dried at 80 °C for about 3 days to obtain the self-standing TpPa-(SO_3_H)_1.5_ COF membrane.

*4. Fabricatio**n of self-standing TpPa-(SO_3_H)_2_ COF membranes*


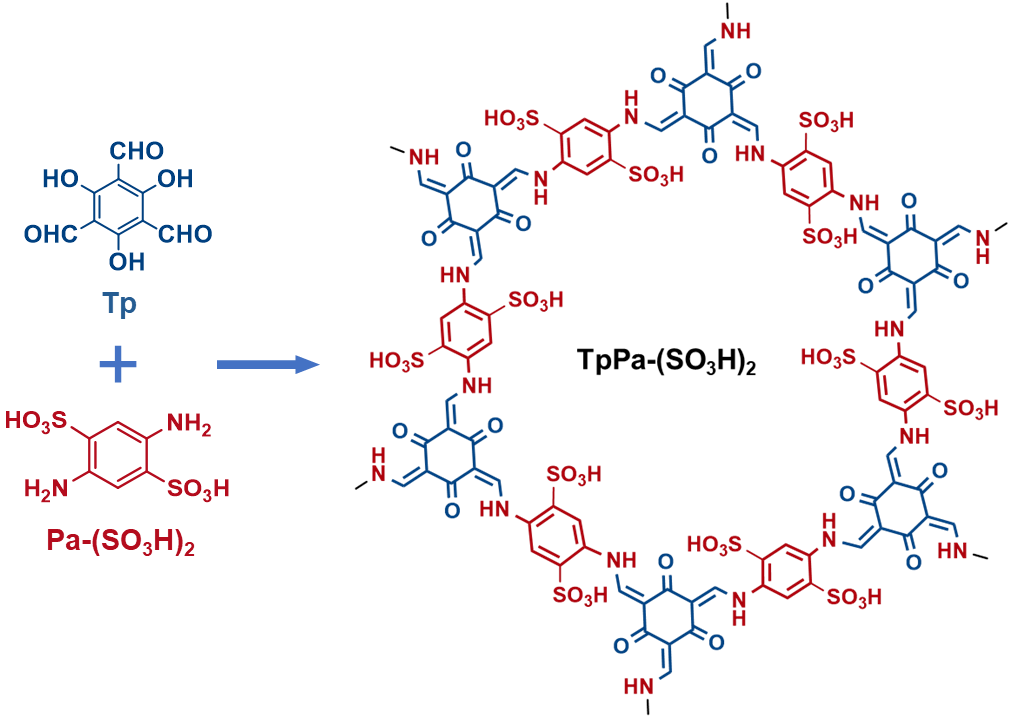


**Scheme S4.** Preparation procedure for TpPa-(SO_3_H)_2_.

Pa-(SO_3_H)_2_ (0.15 mmol, 40.24 mg) and Tp (0.1 mmol, 21.01 mg) were dissolved separately, each in 5 mL DMSO to obtain aldehyde and amine solution, respectively. After dissolution, the aldehyde solution was added dropwise into the amine solution under gentle stirring. Then, the mixture was frozen in liquid N_2_, followed by degassing treatment by three freeze-pump-thaw cycles and heated at 120 °C for 3 days. Subsequently, the resulting mixed solution was poured into a 60 mm diameter Petri dish and was dried at 80 °C for about 3 days to obtain the self-standing TpPa-(SO_3_H)_2_ COF membrane.

**2. Supplementary Figures**


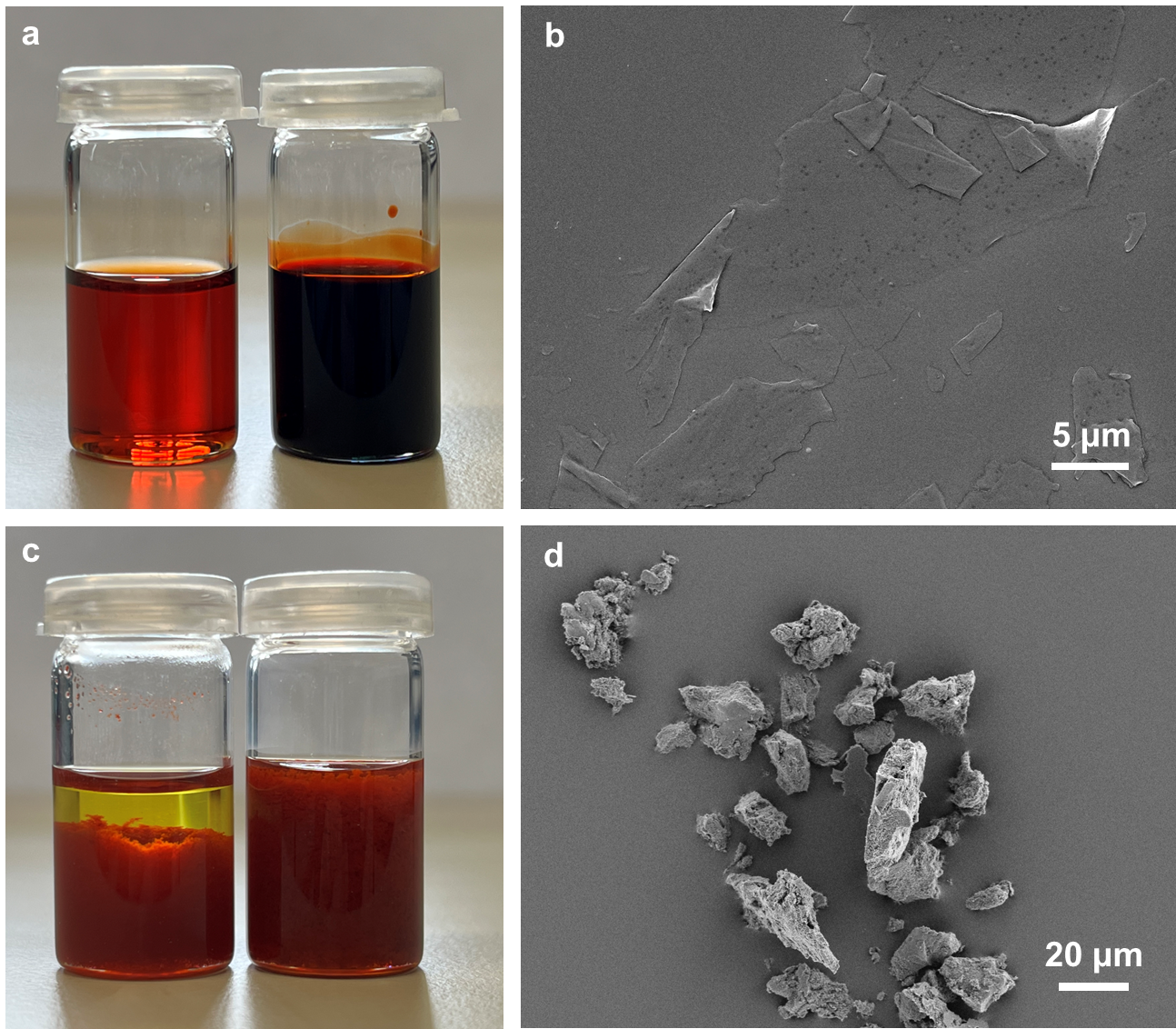


**Figure S1.** (a) Digital photograph of clear and transparent TpPa-(SO_3_H)_1_ colloidal solution (before dilution on the right side and after dilution on the left side). (b) SEM photographs of TpPa-(SO_3_H)_1_ nanosheets. (c) Digital photograph of TpPa suspension (before dilution on the right side and after dilution on the left side) with large amounts of precipitation. (d) SEM photographs of TpPa particles.


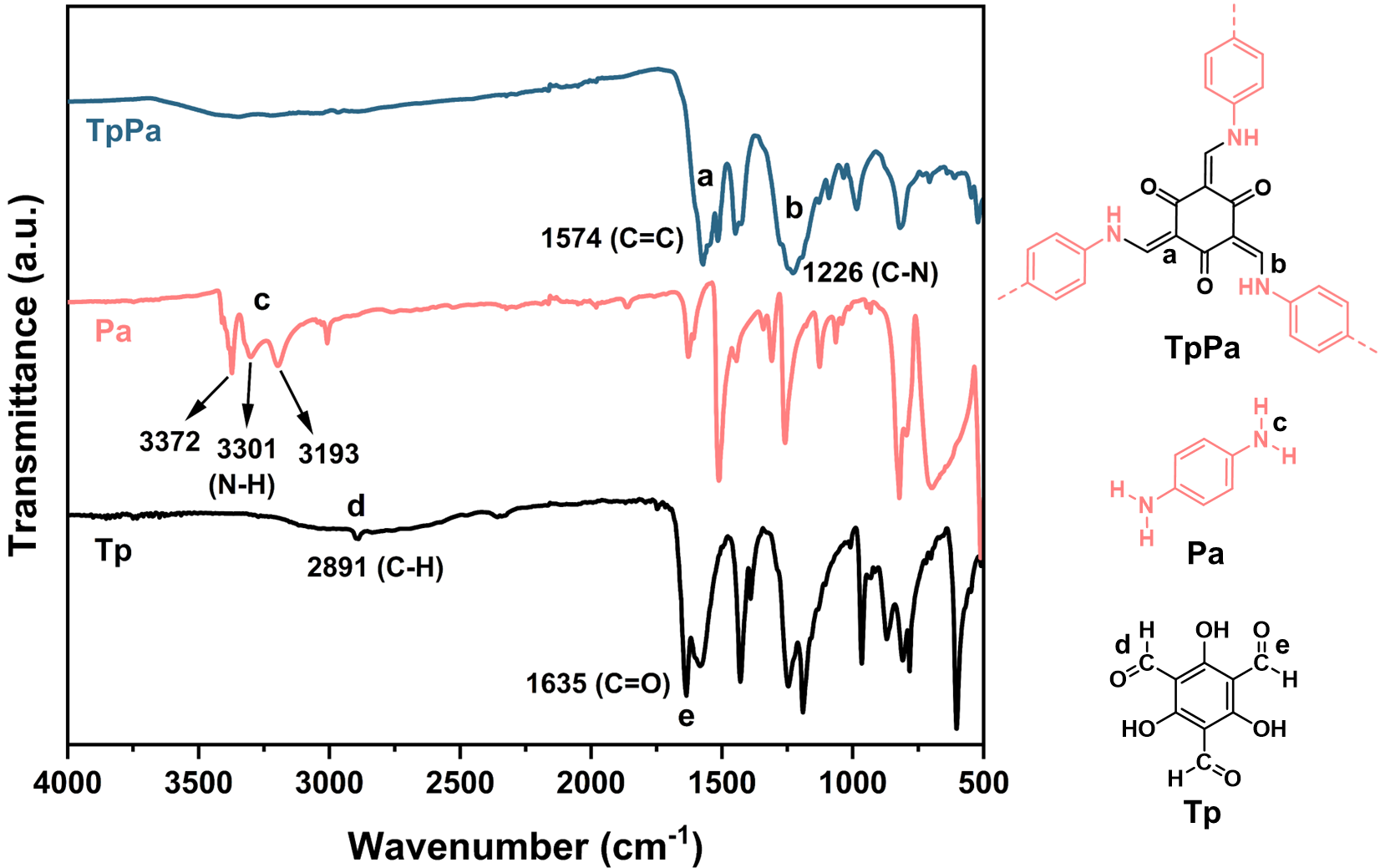


**Figure S2.** FT-IR spectra of Tp, Pa and TpPa COF particles.


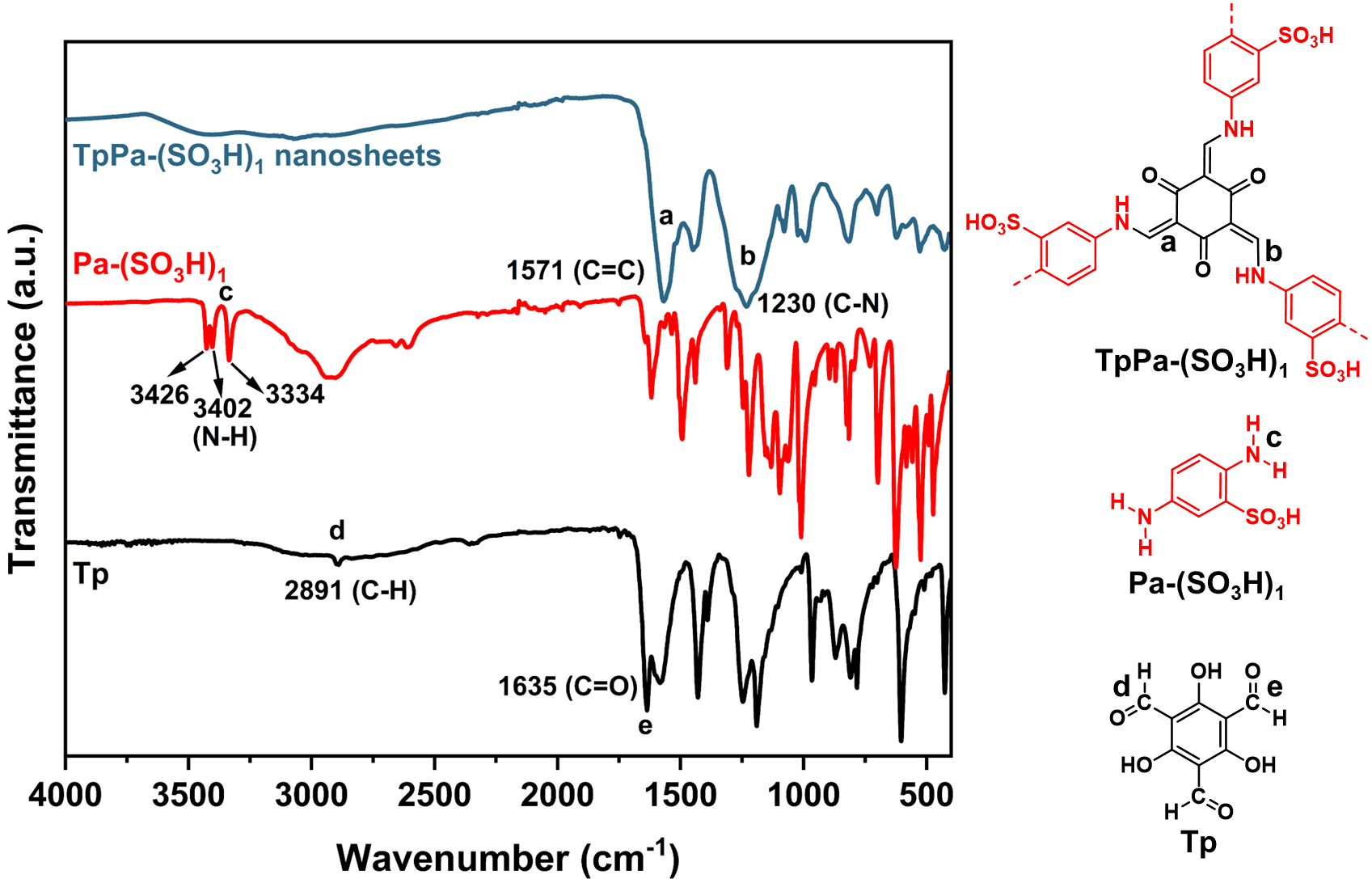


**Figure S3.** FT-IR spectra of Tp, Pa-(SO_3_H)_1_ and TpPa-(SO_3_H)_1_ COF nanosheets.


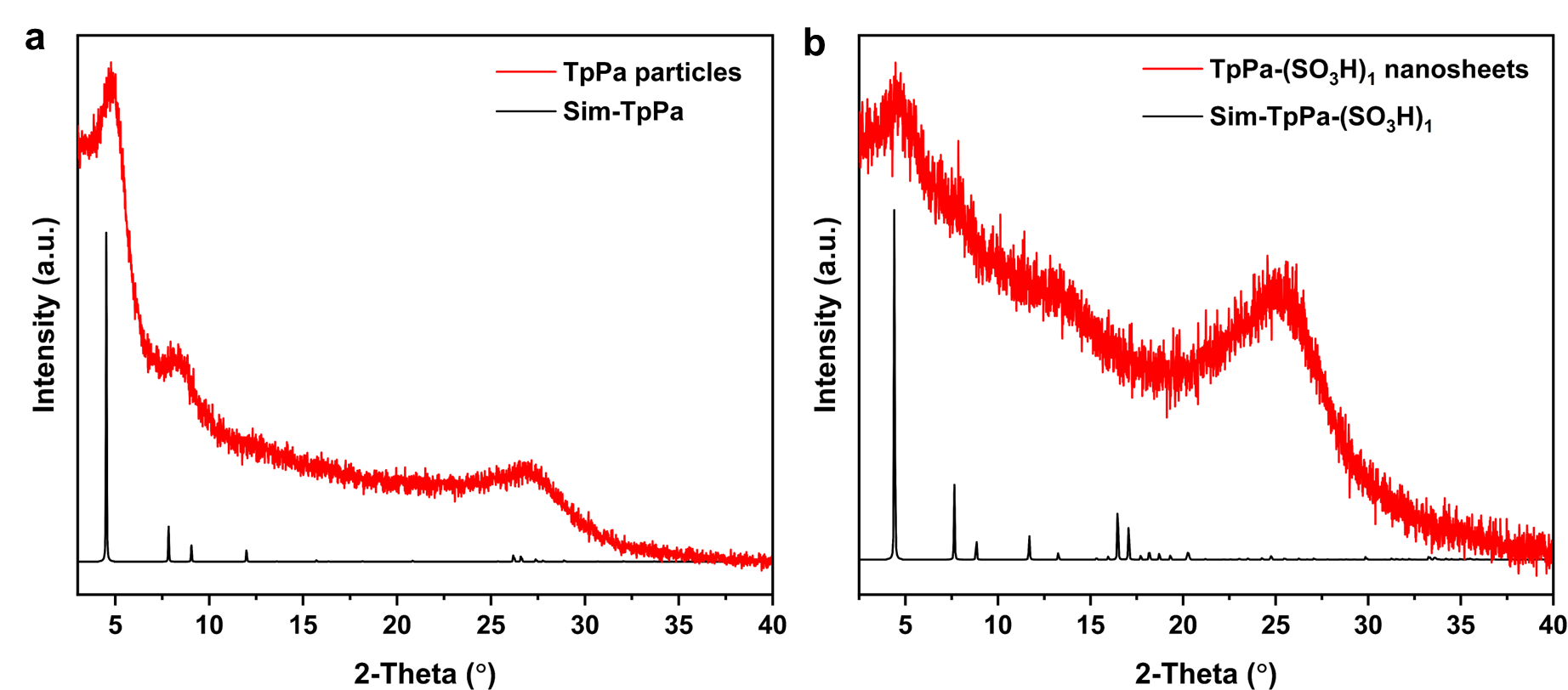


**Figure S4.** (a) XRD patterns of the TpPa particles. The simulated XRD pattern of the TpPa COF (Sim-TpPa) is also shown. (b) XRD patterns of the TpPa-(SO_3_H)_1_ nanosheets. The simulated XRD pattern of the TpPa-(SO_3_H)_1_ COF (Sim-TpPa-(SO_3_H)_1_) is also shown.


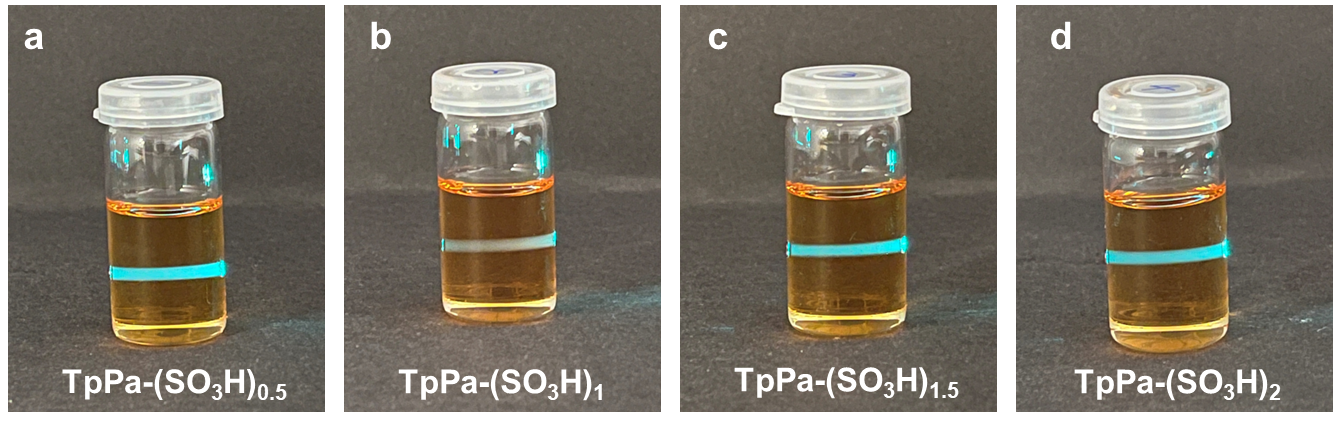


**Figure S5.** The digital photograph of the TpPa-(SO_3_H)_X_ COF nanosheet suspensions, displaying a clear Tyndall effect.

**

**

**Figure S6.** The surface zeta potential of the TpPa-(SO_3_H)_X_ COF nanosheet suspensions.

**
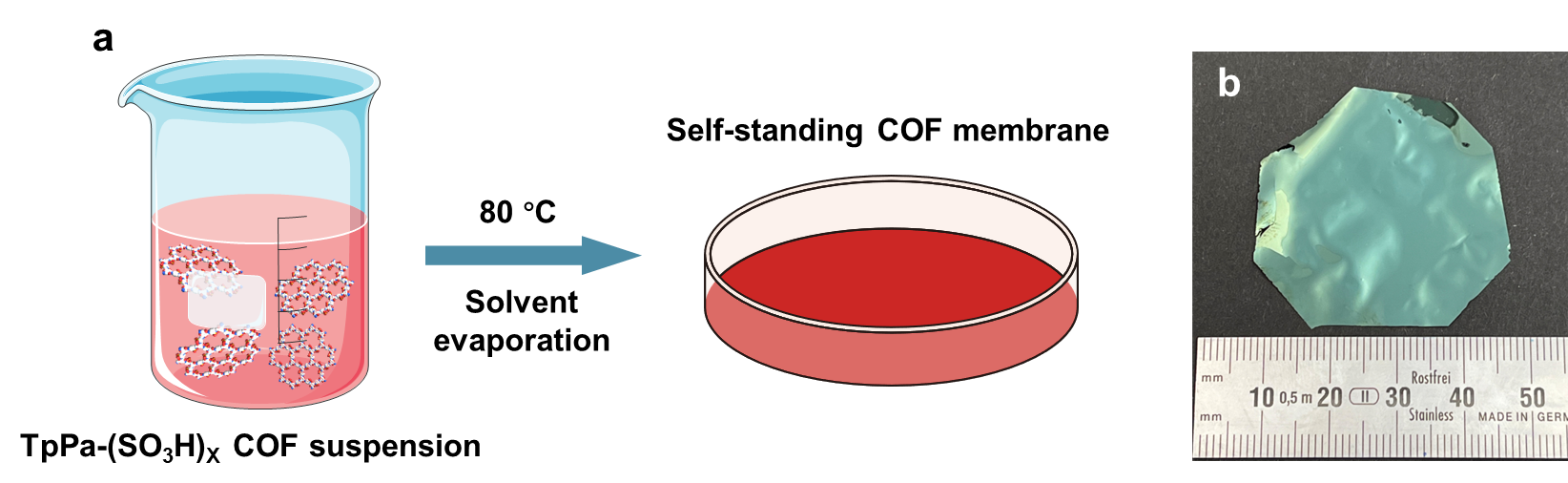
**

**Figure S7.** (a). Schematic illustration of the Self-standing TpPa-(SO_3_H)_x_ COF membranes preparation. (b) Digital photograph of TpPa-(SO_3_H)_1_ membrane.





**Figure S8.** Stress-strain curves of TpPa-(SO_3_H)_X_ membranes.

**Table S1.** Comparison of mechanical properties of polymer-based ion-selective membranes and state-of-the-art self-standing COF membranes.

|  | **Membrane** | **Tensile strength (MPa)** | **Ref.** |
| --- | --- | --- | --- |
| Polymer membranes | PVA-0.9PVBMB | 23.3 | [2] |
|  | PVA-1.2PVBMB | 19.5 |  |
|  | PVA-1.5PVBMB | 17.5 |  |
|  | PVA-1.8PVBMB | 12.4 |  |
|  | PVA-2.1PVBMB | 9.3 |  |
|  | SPX-BP-0.95 | 19.5 | [3] |
|  | QP(SBI/AES)-0 | 48.6 | [4] |
|  | QP(SBI/AES)-0.25 | 46 |  |
|  | QP(SBI/AES)-0.5 | 45.5 |  |
|  | QP(SBI/AES)-0.75 | 36.9 |  |
|  | MTCP-30 | 48.1 | [5] |
|  | MTCP-50 | 44.8 |  |
|  | MTCP-70 | 34.9 |  |
|  | AO-PIM-1 | 52.0 | [6] |
|  | PIM-EA-TB | 42.4 |  |
|  | PIM-1 | 36.9 |  |
|  | SCTF-BP | 29.0 | [7] |
|  | QCTF-BP | 25.0 |  |
|  | *c*PIM-Et | 59.9 | [8] |
|  | *c*PIM-Ph | 60.0 |  |
|  | *c*PIM-BP | 34.0 |  |
|  | PIM-SBF | 70.9 | [9] |
|  | *s*PIM-SBF-0.98 | 45.3 |  |
|  | *s*PIM-SBF-1.40 | 46.4 |  |
|  | *s*PIM-SBF-1.67 | 39.1 |  |
|  | *s*PIM-SBF-1.86 | 29.8 |  |
| COF membranes | iCOFM | 20.2 | [10] |
|  | PTSA@TpAzo COFMs | 16.3 | [11] |
|  | COF-42 | 0.27 | [12] |
|  | COF-200 | 0.64 | [13] |
|  | TpBD-(SO_3_H)_2_ iCOFMs | 20 | [14] |
| COF membranes | TpPa-1 | 21.19 | [15] |
|  | COF-QA-2 | 52 | [16] |
|  | COF-QA-4 | 49 |  |
|  | COF-QA-6 | 53 |  |
|  | COF-QA-EO | 50 |  |
|  | TpPa-SO_3_H | 39.3 | [17] |
|  | TpPa-1 | 20.8 | [18] |
|  | TpBD | 21.2 |  |
|  | TpHZ | 14.6 |  |
|  | TpBD(OH)_2_ | 25.3 |  |
|  | TpDa | 28 | [19] |
|  | COF-SQA | 37 | [20] |
|  | TpPa-(SO_3_H)_0.5_ | 89.7 | This work |
|  | TpPa-(SO_3_H)_1_ | 81.6 |  |
|  | TpPa-(SO_3_H)_1.5_ | 69.9 |  |
|  | TpPa-(SO_3_H)_2_ | 65.2 |  |


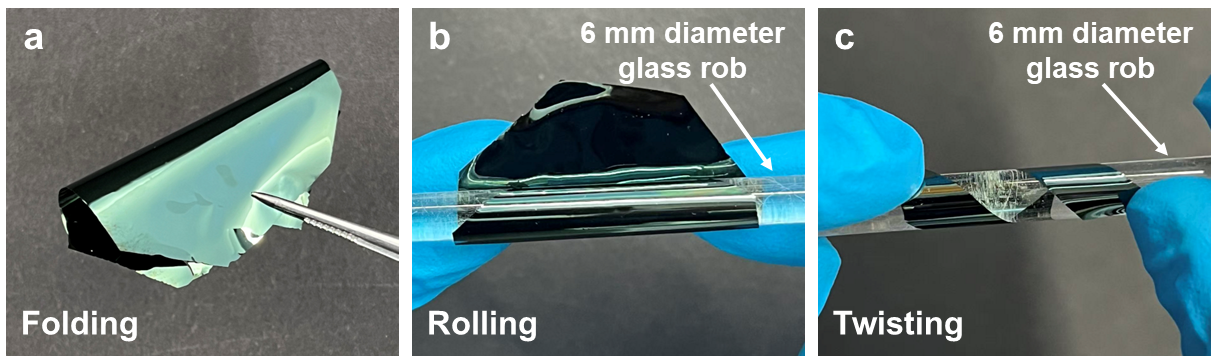


**Figure S9.** TpPa-(SO_3_H)_1_ membrane (a) folded, (b) rolled, (c) twisted on ≈6 mm diameter glass rod.

**
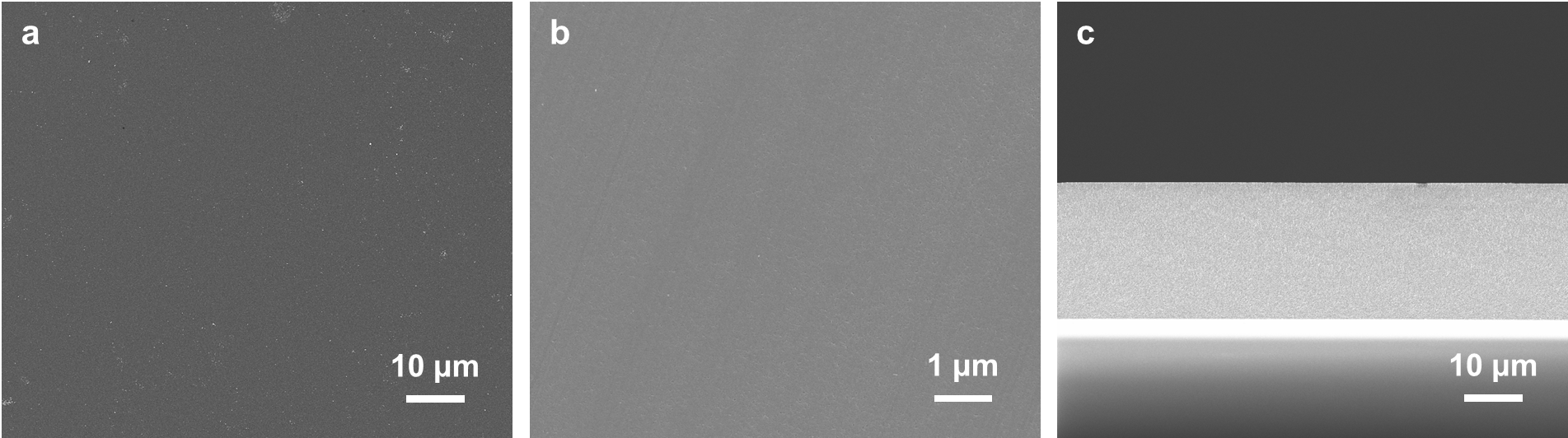
**

**Figure S10.** (a-b) Top-view SEM images, (c) cross-section view SEM images of TpPa-(SO_3_H)_0.5_ COF membranes.


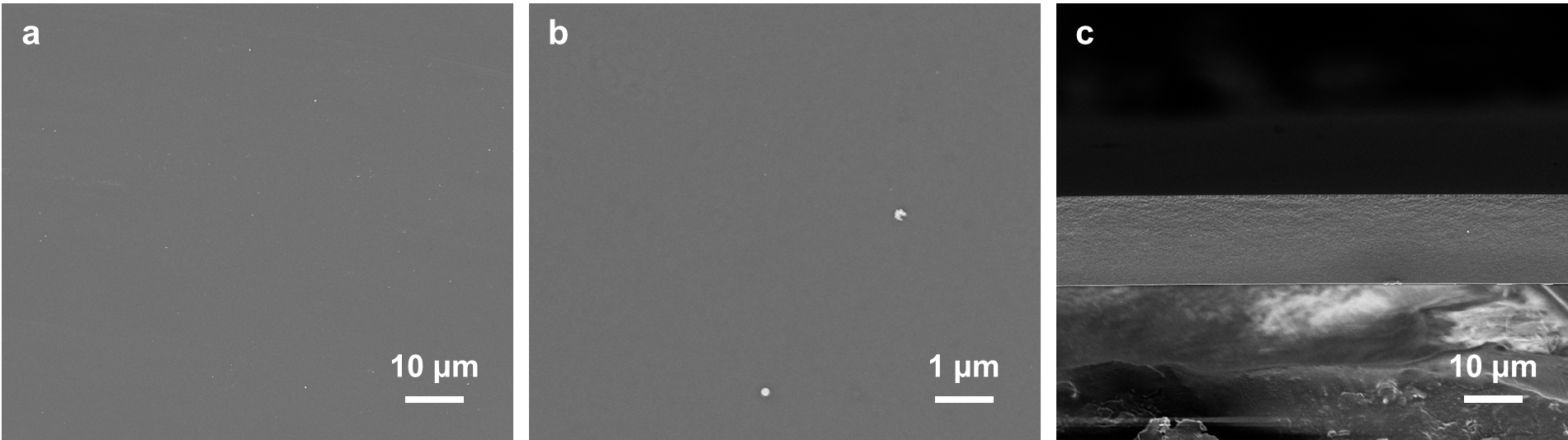


**Figure S11.** (a-b) Top-view SEM images, (c) cross-section view SEM images of TpPa-(SO_3_H)_1.5_ COF membranes.


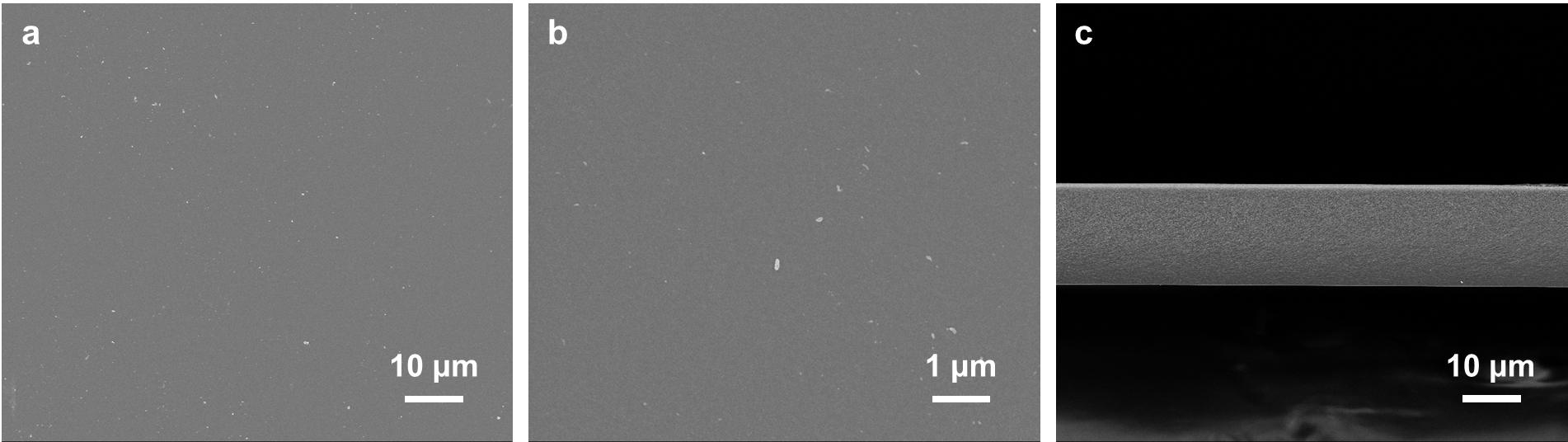


**Figure S12.** (a-b) Top-view SEM images, (c) cross-section view SEM images of TpPa-(SO_3_H)_2_ COF membranes.





**Figure S13.** FT-IR spectra of Tp, Pa, Pa-(SO_3_H)_1_, TpPa-(SO_3_H)_0.5_ and TpPa-(SO_3_H)_1_.





**Figure S14.** FT-IR spectra of Tp, Pa-(SO_3_H)_1_, Pa-(SO_3_H)_2_, TpPa-(SO_3_H)_1.5_ and TpPa-(SO_3_H)_2_.


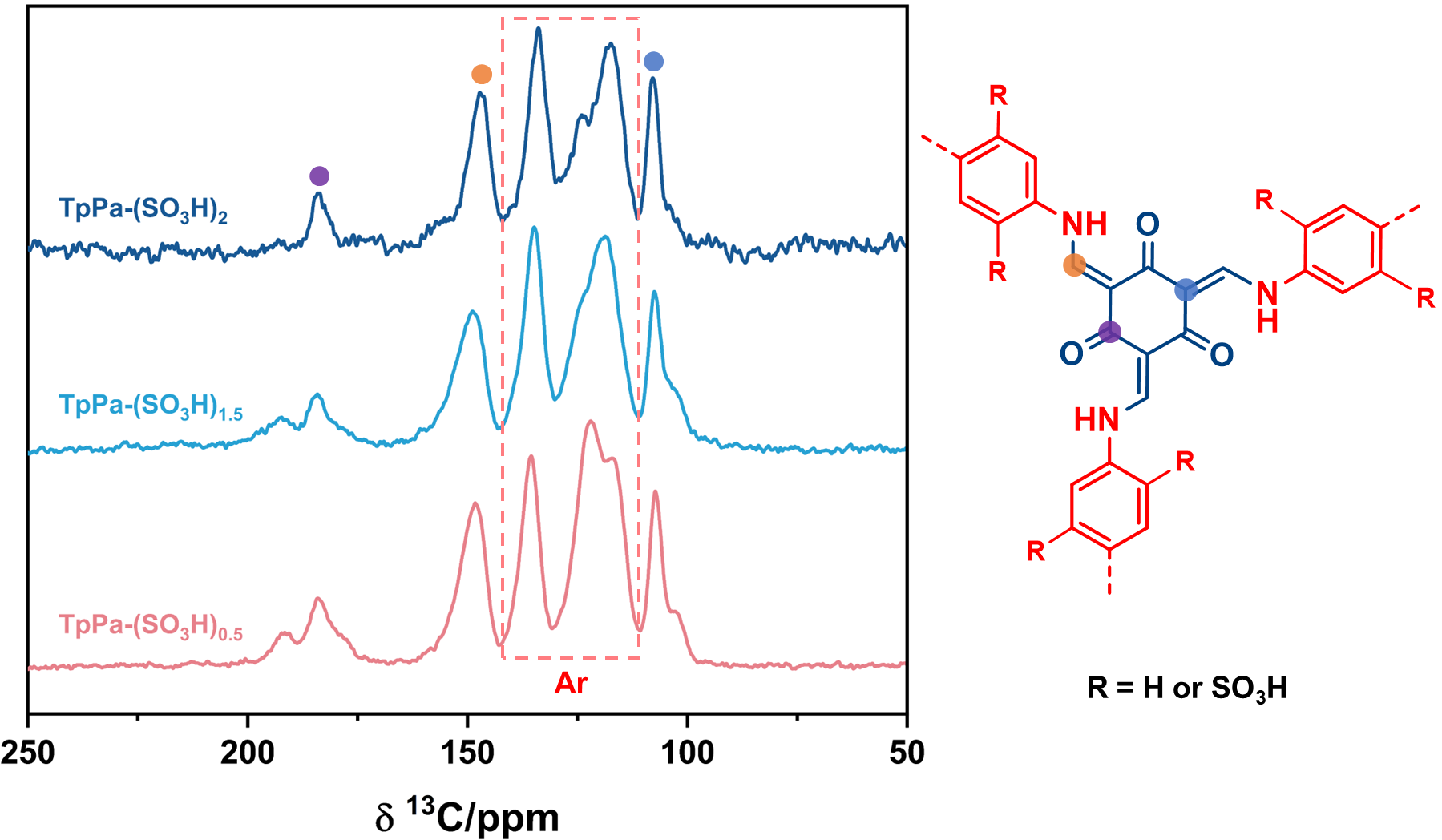


**Figure S15.** The solid-state ^13^C NMR of TpPa-(SO_3_H)_x_ COF membranes.

**Table S2.** The weight percentages (wt.%) of sulfur in the self-standing COF-(SO_3_H)_X_ membranes and the COF-(SO_3_H)_X_ powder samples synthesized via a solvothermal method.

|  | **Theoretical values (wt. %)** | **Elemental analysis (wt. %)** | | | | | |
| --- | --- | --- | --- | --- | --- | --- | --- |
|  |  | **COF membrane** | | | **COF powder** | | |
| TpPa-(SO_3_H)_0.5_ | 6.36 | 6.62 | 6.54 | 6.52 | 6.37 | 6.42 | 6.48 |
| TpPa-(SO_3_H)_1_ | 10.97 | 8.66 | 8.76 | 8.73 | 10.61 | 10.70 | 10.64 |
| TpPa-(SO_3_H)_1.5_ | 14.48 | 9.74 | 9.79 | 9.64 | 11.19 | 11.06 | 10.98 |
| TpPa-(SO_3_H)_2_ | 17.23 | 10.03 | 9.97 | 10.09 | 11.23 | 11.60 | 11.18 |

**
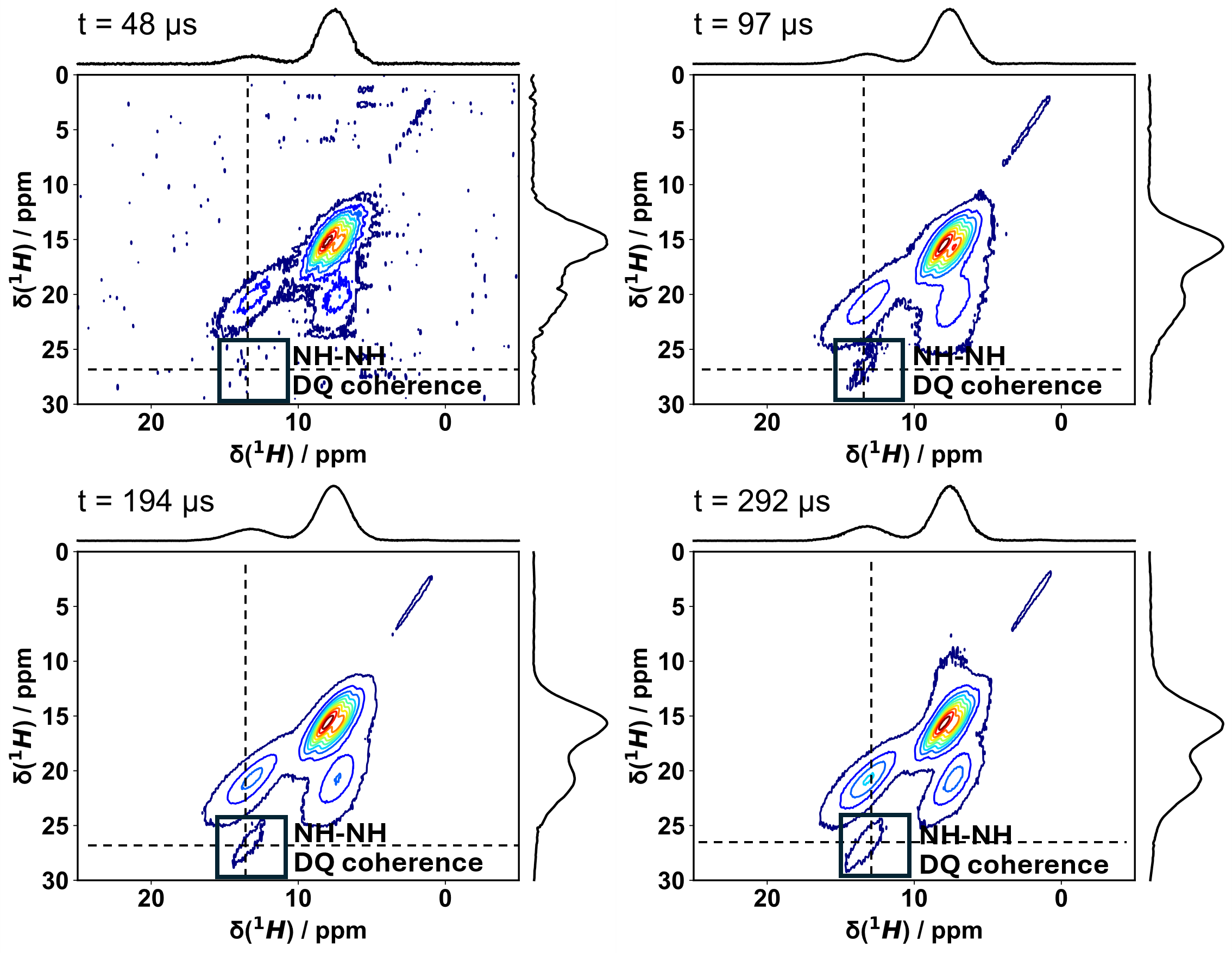
**

**Figure S16.** ^1^H-^1^H DQ-SQ spectra of TpPa-(SO_3_H)_0.5_ COF membranes with various recoupling-times (recoupling time = build-up time + reconversion time), highlighted NH-NH DQ coherence.

**
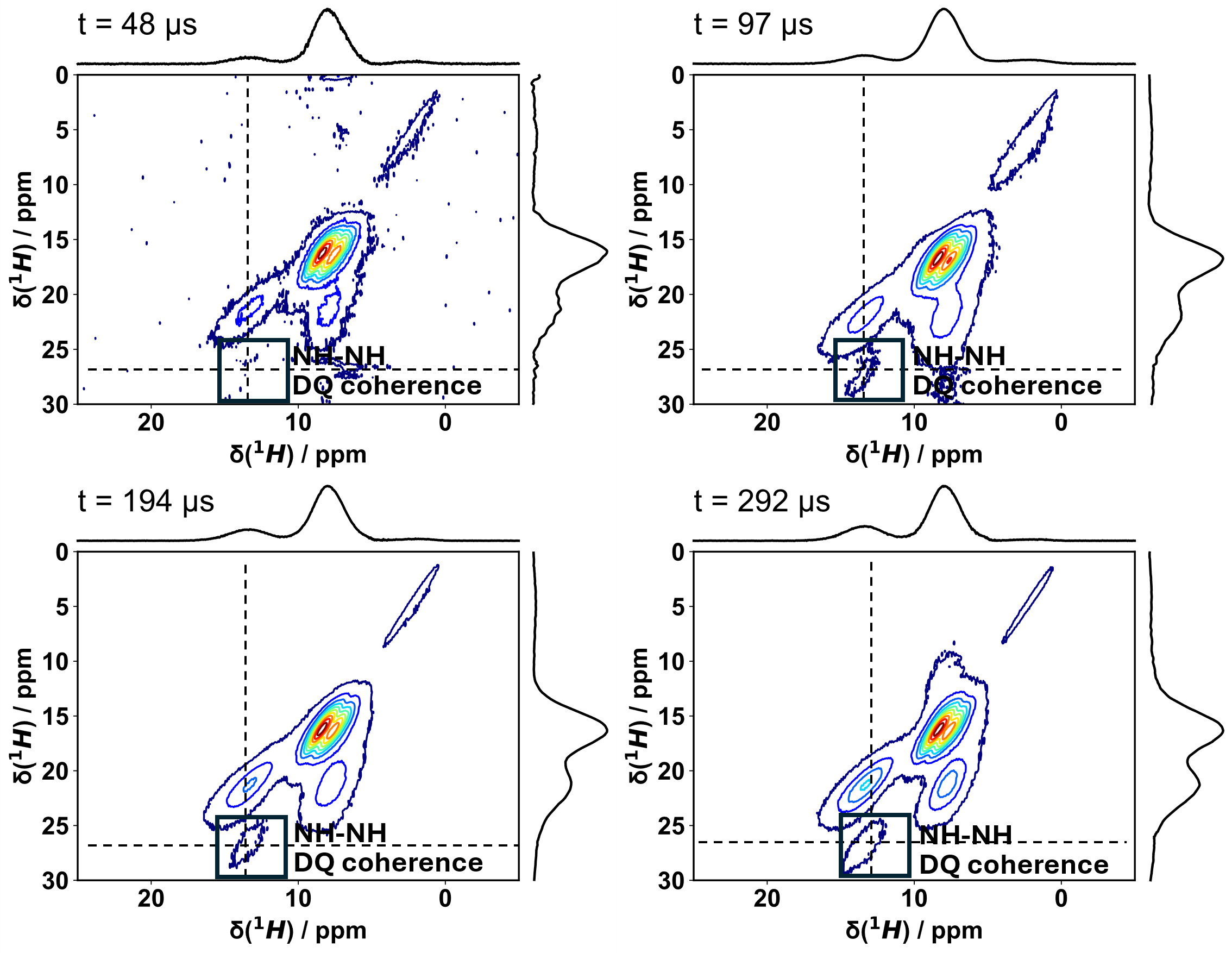
**

**Figure S17.** ^1^H-^1^H DQ-SQ spectra of TpPa-(SO_3_H)_1_ COF membranes with various recoupling-times (recoupling time = build-up time + reconversion time), highlighted NH-NH DQ coherence.

**
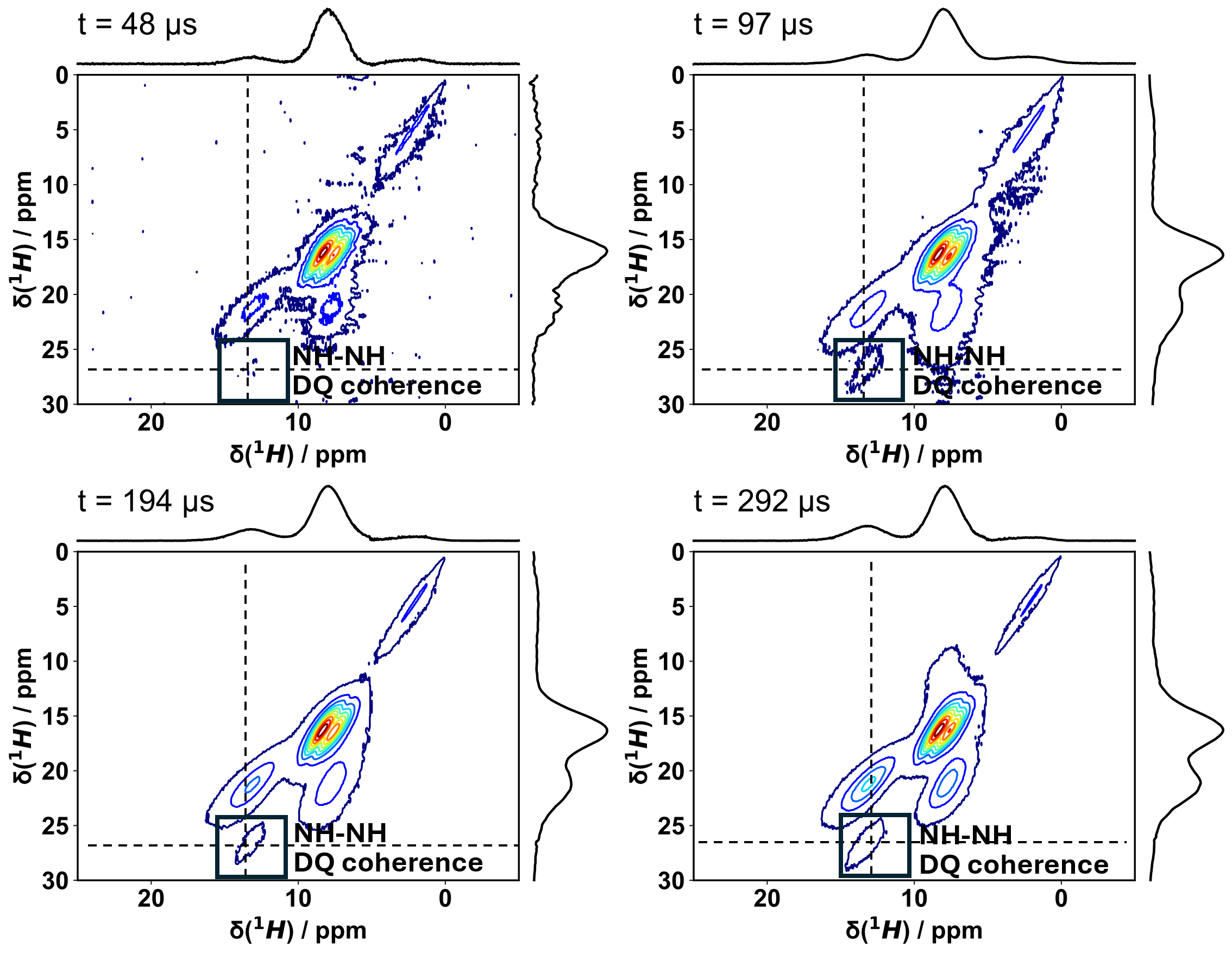
**

**Figure S18.** ^1^H-^1^H DQ-SQ spectra of TpPa-(SO_3_H)_1.5_ COF membranes with various recoupling-times (recoupling time = build-up time + reconversion time), highlighted NH-NH DQ coherence.

**
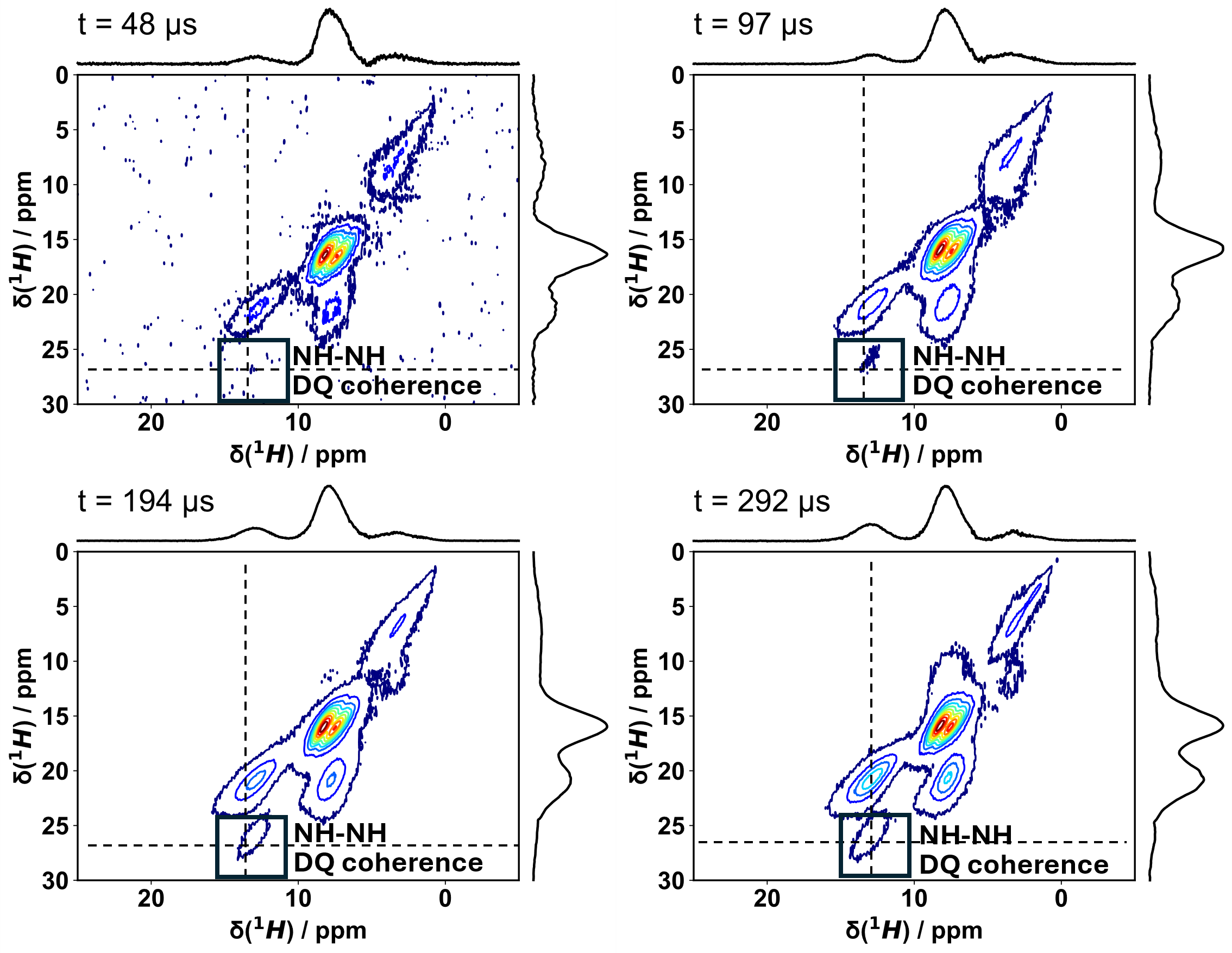
**

**Figure S19.** ^1^H-^1^H DQ-SQ spectra of TpPa-(SO_3_H)_2_ COF membranes with various recoupling-times (recoupling time = build-up time + reconversion time), highlighted NH-NH DQ coherence.


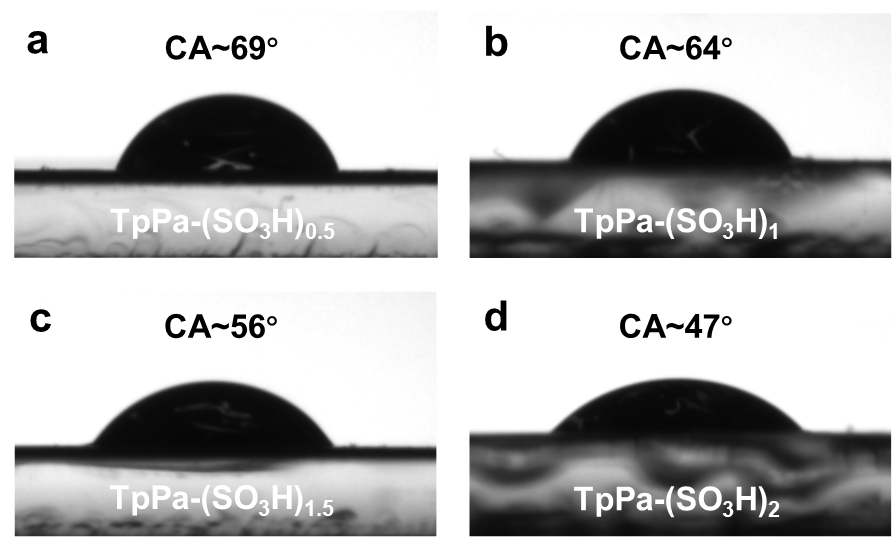


**Figure S20.** Water contact angle of TpPa-(SO_3_H)_X_ membranes.


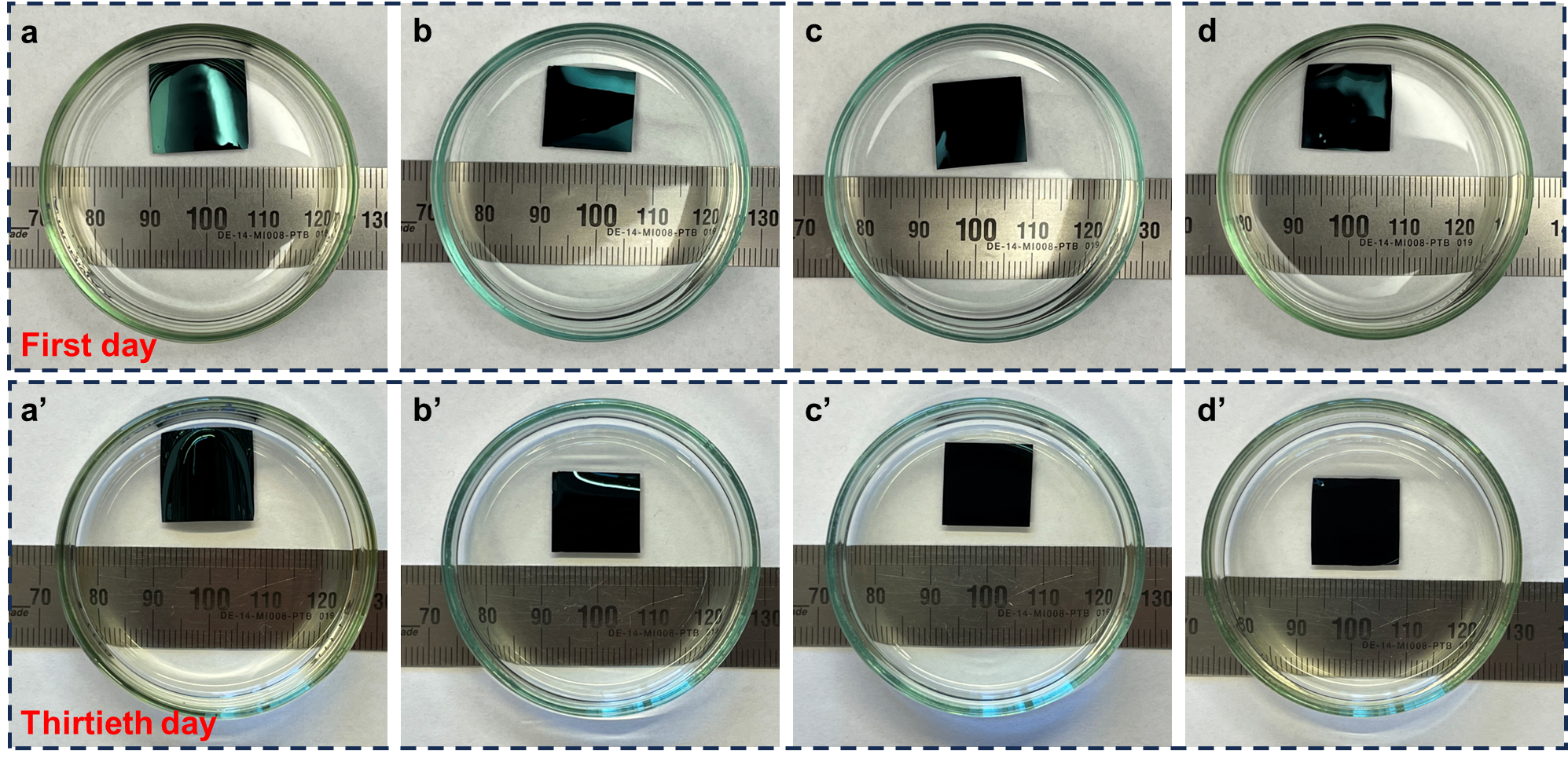


**Figure S21.** The digital photographs of (a) TpPa-(SO_3_H)_0.5_, (b) TpPa-(SO_3_H)_1_, (c) TpPa-(SO_3_H)_1.5_ and (d)TpPa-(SO_3_H)_2_ membranes immersed in deionized water on the first day, and the digital photographs of (a’) TpPa-(SO_3_H)_0.5_, (b’) TpPa-(SO_3_H)_1_, (c’) TpPa-(SO_3_H)_1.5_ and (d’) TpPa-(SO_3_H)_2_ membranes immersed in deionized water for 30 days.

**Measurement of transmembrane conductance**

The transmembrane transport behaviour of ions was assessed by recording current-voltage (*I*-*V*) curves. The COF membrane was placed between two diffusion cells (4 cm^3^) with 1 mm aperture, and both cells were filled with the same concentration of KCl solution ranging from 0.01 mM to 3 M. A pair of Ag/AgCl electrodes was embedded in two chambers, which were then connected to a source meter (KEITHLEY, 2450 SourceMeter^®^). *I*-*V* curves were recorded by applying a scanning voltage (-0.2 V, 0.2 V) in 4 mV steps. Conductance values were derived from the slope of the resulting *I*-*V* curve.


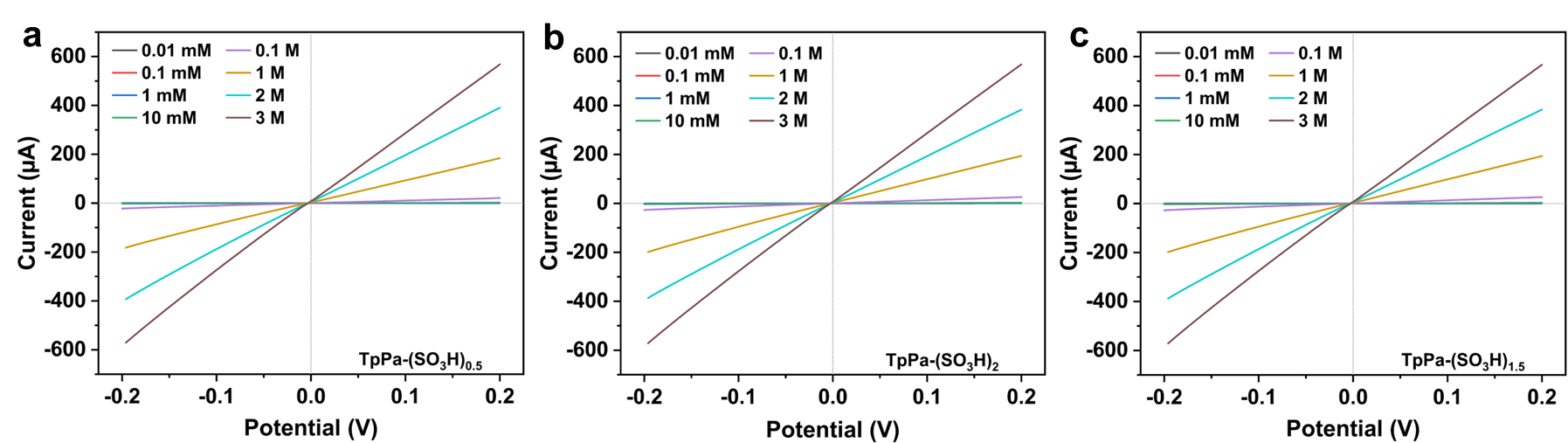


**Figure S22.** *I*–*V* curves of the TpPa-(SO_3_H)_0.5_ membrane, TpPa-(SO_3_H)_1.5_ membrane, and TpPa-(SO_3_H)_2_ membrane recorded in KCl solutions with concentrations ranging from 0.01 mM to 3 M.

**Measurement of ion transference number**

**Step 1:** To characterize the ion-selective transport through charged nanochannel in COF membranes, *I*-*V* curves were collected using a wide range of KCl concentration differences across the TpPa-(SO_3_H)_X_ membranes. The COF membrane was placed between two diffusion cells (4 cm^3^) with 5 mm aperture, and both cells were filled with the different concentration of KCl solution. The KCl concentration was held constant at 0.1 mM in one reservoir (*C*_low_), while in the opposing reservoir (*C*_high_), it was progressively increased from 1 mM to 0.1 M. *I*-*V* curves were recorded by applying a scanning voltage (-0.4 V, 0.4 V) in 8 mV steps. The open-circuit voltage (*V*_oc_) and short-circuit current (*I*_sc_) were obtained from the interception of the *I*-*V* curves at zero voltage and current, respectively.


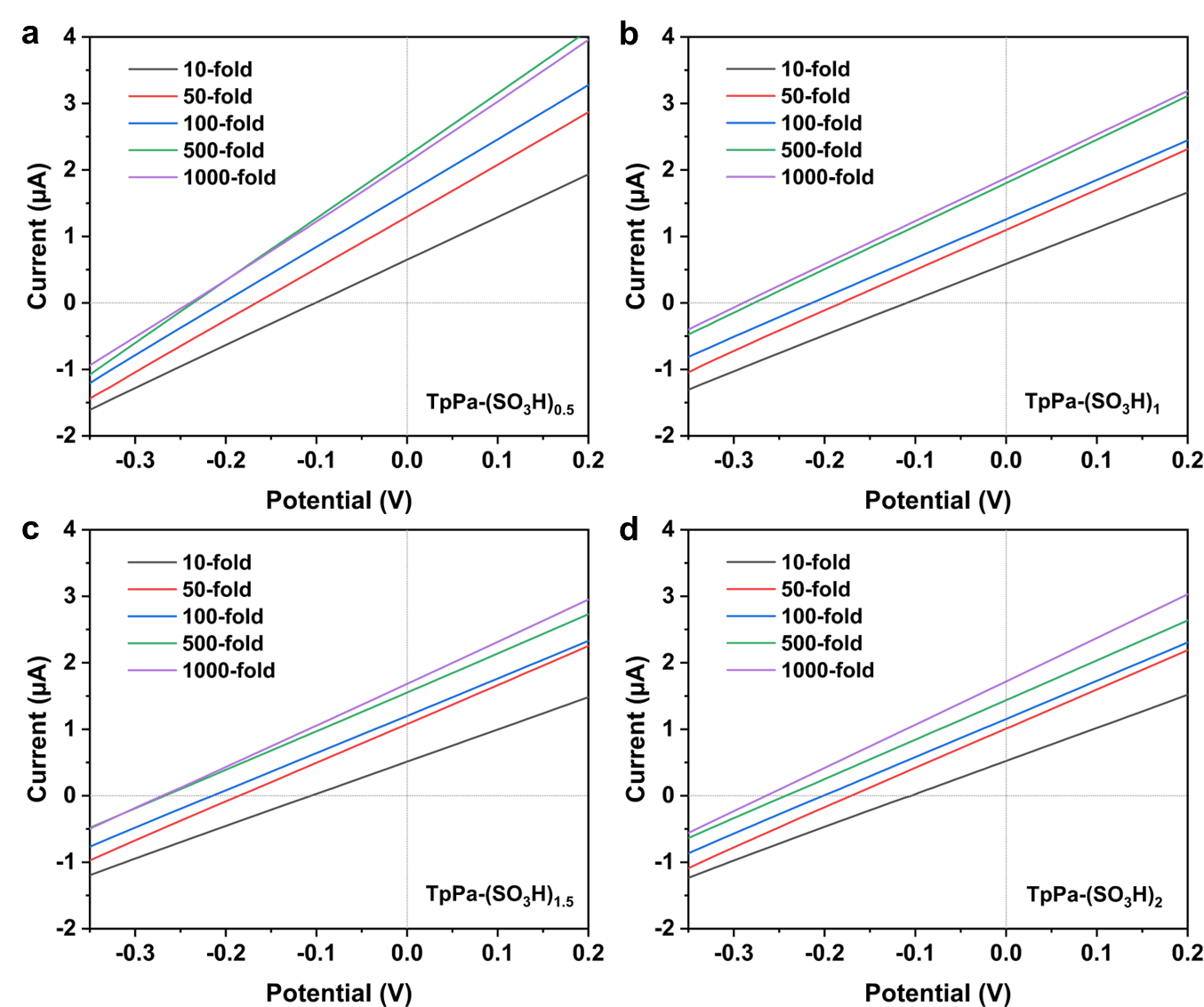


**Figure S23.** The *I*-*V* curves of the TpPa-(SO_3_H)_X_ membranes under different KCl concentration difference.


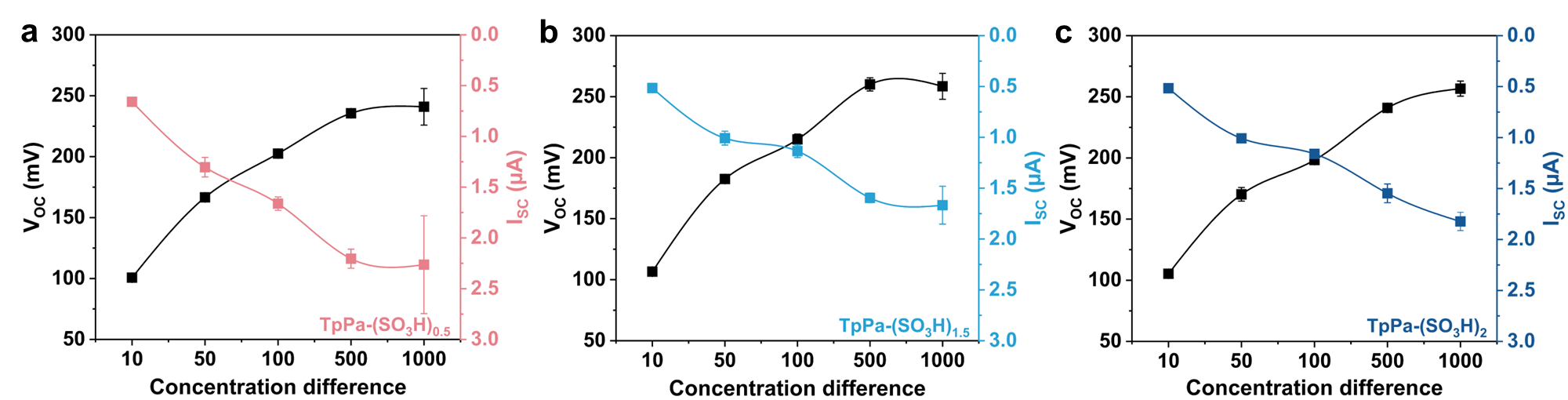


**Figure S24.** Plots of the recorded *V*_OC_ and *I*_SC_ versus KCl concentration difference for the TpPa-(SO_3_H)_0.5_ membrane, TpPa-(SO_3_H)_1.5_ membrane, and TpPa-(SO_3_H)_2_ membrane.

**Step 2:** The *V*_oc_ consists of the redox potential (*E*_redox_) and the diffusion potential (*E*_diff_), however, the *E*_redox_ is derived from external chemical energy and therefore the cation transfer number (*t*_+_) can be calculated by the following equation.

$$t_{+}\boldsymbol{=}\frac{1}{2}\boldsymbol{(}\frac{\left| E_{diff} \right|}{\frac{RT}{F}\ln\frac{\gamma_{high}C_{high}}{\gamma_{low}C_{low}}}\boldsymbol{+}1\boldsymbol{)} \boldsymbol{(}S1\boldsymbol{)}$$

$$E_{diff}\boldsymbol{=}V_{oc}\boldsymbol{-}E_{redox} (S2)$$

Where *C*_high_, *C*_low_, *γ*_high_ and *γ*_low_ are the concentration and activity coefficient of the high and low concentration solutions respectively; *R*, *F* and *T* refer to the universal gas constant, Faraday constant and absolute temperature, respectively.

**Table S3.** Values for the activity coefficients of potassium chloride in water at 25 °C.^[21]^

| Concentration | 0.1 mM | 1 mM | 5 mM | 10 mM | 50 mM | 0.1 M |
| --- | --- | --- | --- | --- | --- | --- |
| Activity coefficient (*γ*) | 0.998 | 0.965 | 0.927 | 0.901 | 0.816 | 0.768 |

**Redox potential measurement of Ag/AgCl electrodes**

A COF membrane with cation selectivity was placed between two saline waters with asymmetric concentrations. Under a concentration gradient, cations cross the selective membrane and accumulate on the low salinity side, while anions remain on the high salinity side, and in order to maintain electroneutrality, the electrode undergoes a redox reaction. However, the redox potential (denoted as *E*_redox_) is derived from external chemical energy, not the osmotic energy itself. Therefore, in order to accurately quantify the osmotic energy the contribution of the redox potential needs to be eliminated. To ensure that the measured potentials were only from asymmetric redox reactions occurring at the electrodes, the COF membranes were replaced with PAN membranes without selectivity. The redox potential of the electrodes under different concentration gradients was recorded (The KCl concentration was held constant at 0.1 mM in the low concentration side, while in the opposing reservoir, it was progressively increased from 0.5 mM to 1 M.). As shown in Figure S20, the experimental measurements are in good agreement with the calculated values of the Nernst equation.

$$E=E_{Ag/AgCl}^{^{\circ}}+\frac{RT}{nF}\ln[{Cl}^{-}] (S3)$$

Where *E*, *E°_Ag/AgCl_*, and *[Cl^-^]* are electrode potential and standard electrode potential and chloride ion concentration respectively; *R*, *T*, *n* and *F* refer to the universal gas constant, absolute temperature, number of transferred electrons and Faraday constant, respectively. The value of *E°_Ag/AgCl_* is 0.222 V.^[21]^





**Figure 25.** Comparison of experimental and theoretical redox potentials of Ag/AgCl electrodes at different KCl concentration gradients.

**Measurement of osmotic energy harvesting**

The COF membrane was placed between two diffusion cells (4 cm^3^) with 0.1 mm aperture, and the cells were filled with electrolyte solutions with different concentration. A pair of Ag/AgCl electrodes was embedded in two chambers, which were then connected to a source meter (KEITHLEY, 2450 SourceMeter^®^). *I*-*V* curves were recorded by applying a scanning voltage (-0.2 V, 0.2 V) in 4 mV steps. The open-circuit voltage (*V*_oc_) and short-circuit current (*I*_sc_) were obtained from the interception of the I-V curves at zero voltage and current, respectively. The maximum output power density can be calculated by the following formula.

$$P_{max}=\frac{I_{sc}V_{oc}}{4S} (S4)$$

where *P*_max_ and S refer to the maximum output power density and the effective testing area (0.0785 mm^2^) of the COF membrane, respectively.


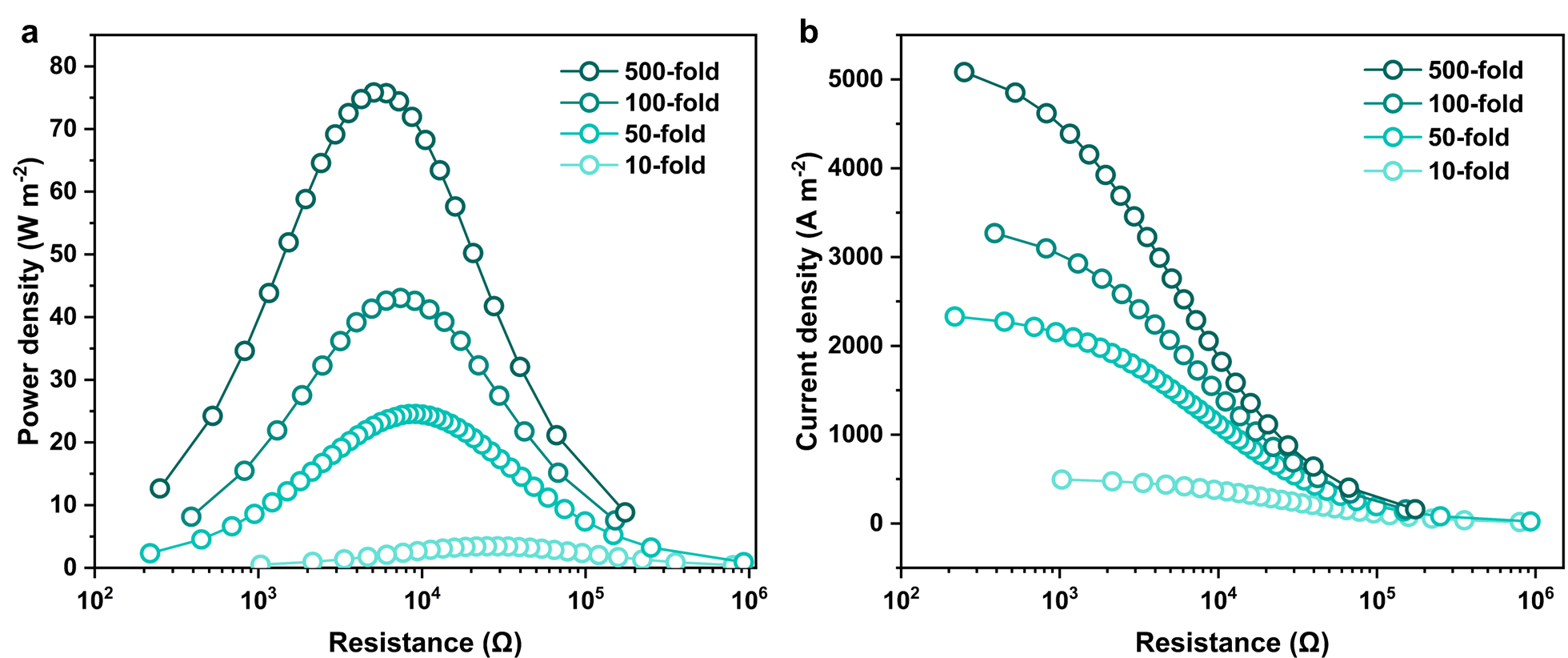


**Figure S26.** (a) The output power density and (b) current density of RED system based on TpPa-(SO_3_H)_1_ membrane at different concentration gradients. The low-salinity NaCl solution is fixed at 0.01 M, and high-salinity is varied from 0.1 M to 5 M.


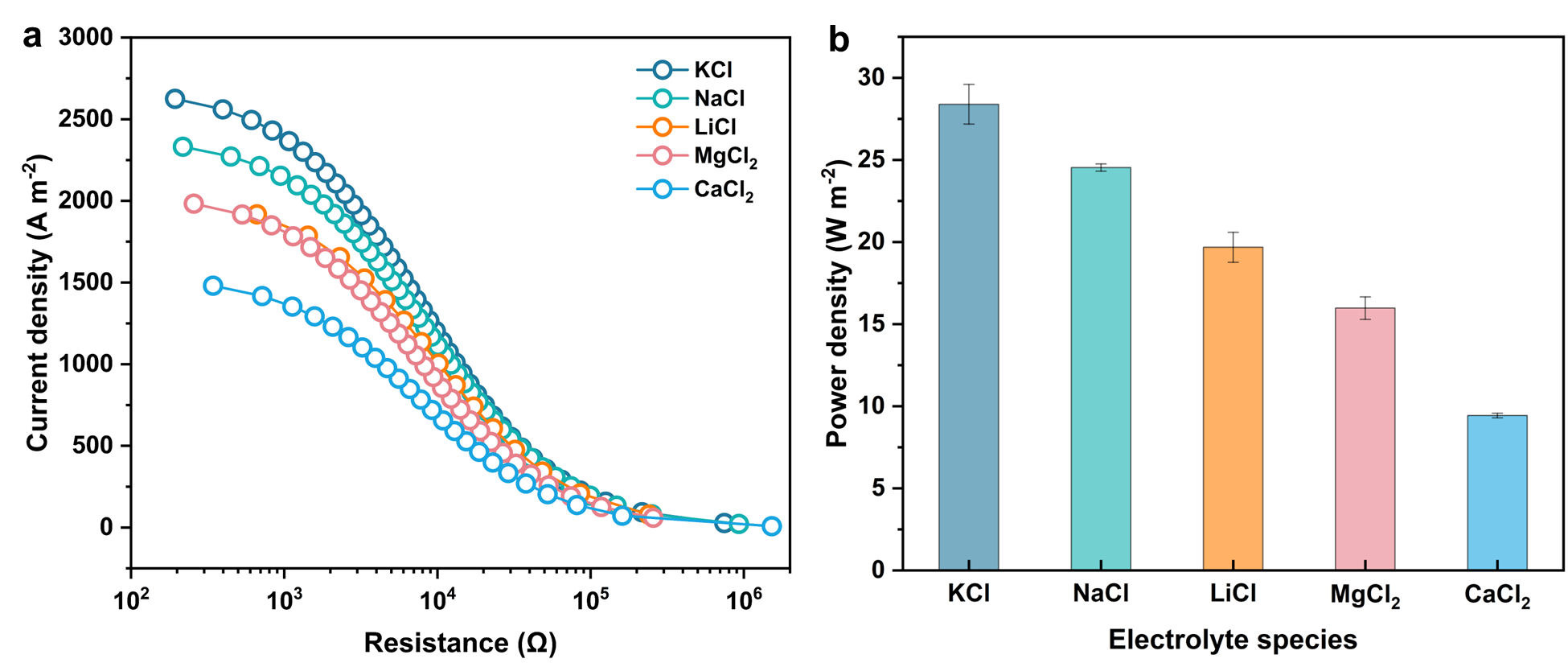


**Figure S27.** (a) The output current density at different electrolytes of the TpPa-(SO_3_H)_1_ membranes as a function of the external load resistance under a 50-fold salinity gradient (0.5 M/0.01 M). (b) The maximum output power density at different electrolytes of RED system based on TpPa-(SO_3_H)_1_ membrane under a 50-fold salinity gradient (0.5 M/0.01 M).

**Table S4.** Ion Diffusion coefficients in bulk solution.^[21]^

| **Ion** | **Diffusion coefficient**  **(m^2^ s^-1^)** |
| --- | --- |
| K^+^ | 1.957×10^-9^ |
| Cl^-^ | 2.032×10^-9^ |
| Na^+^ | 1.334×10^-9^ |
| Li^+^ | 1.029×10^-9^ |
| Ca^2+^ | 0.792×10^-9^ |
| Mg^2+^ | 0.706×10^-9^ |





**Figure S28.** The output current density at different pH values of the TpPa-(SO_3_H)_1_ membrane as a function of the external load resistance under a salinity gradient of 0.5 M/0.01 M NaCl.

**Long-term stability test**

The TpPa-(SO_3_H)_1_ COF membrane was mounted between two compartments of a diffusion cell with 0.1 mm aperture, filled with 0.5 M and 0.01 M NaCl solutions respectively. A pair of Ag/AgCl electrodes were immersed directly into the solutions and connected to an electrochemical workstation (Interface 1010E, Gamry). The voltage-time (*V*-*t*) curve of the reverse electrodialysis (RED) system was continuously recorded for 10 h without compensating electrolytes to characterize voltage decay behavior.


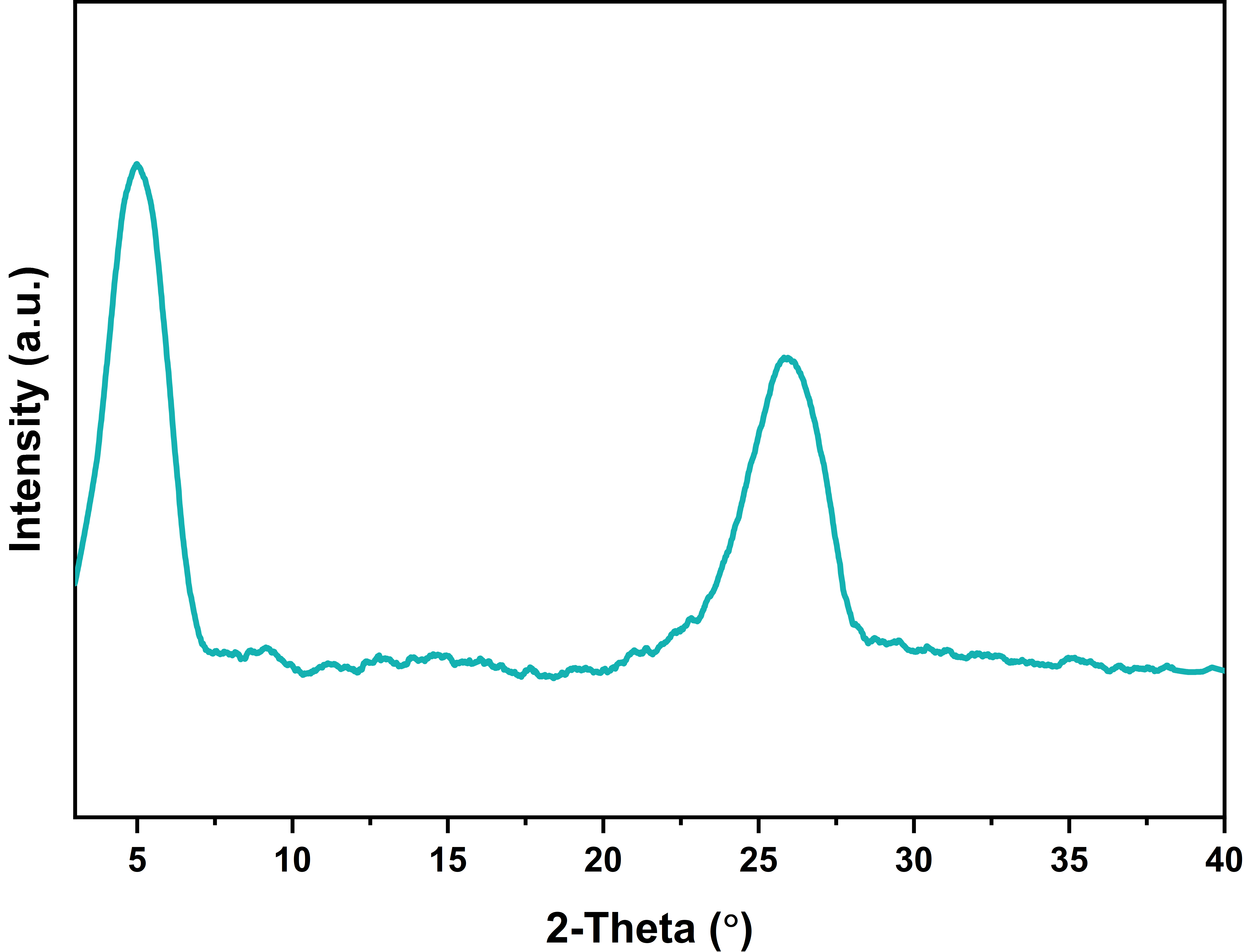


**Figure S29.** PXRD pattern of the free-standing TpPa-(SO_3_H)_1_ COF membrane after being soaked in 0.5 M NaCl aqueous solution for 2 days.


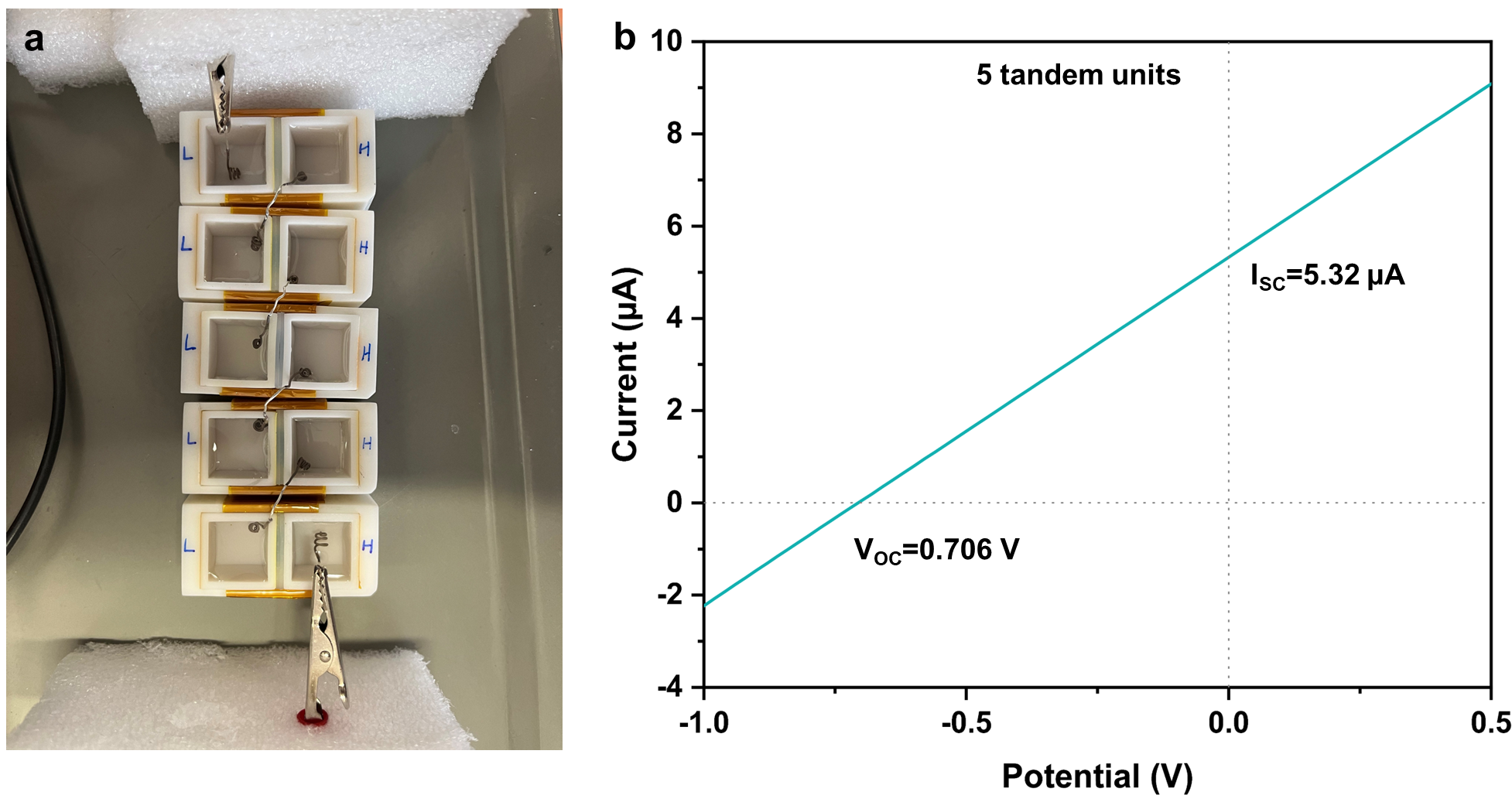


**Figure S30.** (a) Digital photograph of five TpPa-(SO_3_H)_1_-based osmotic energy harvesting system connected in tandem and (b) the corresponding *I*-*V* curve. Each cell is divided into two compartments by a TpPa-(SO_3_H)_1_ COF membrane, filled with 0.01 M and 0.5 M NaCl aqueous solutions respectively, and connected using Ag/AgCl electrodes.

**Table S5.** Osmotic energy harvesting performance of representative RED systems in the literature.

| **Materials** | **System (Aqueous)** | **Testing area (mm^2^)** | **Power density (W m^-2^)** | | **Ref.** |
| --- | --- | --- | --- | --- | --- |
| SAMM-2@AAO | 0.5/0.01 M NaCl | 0.004 | | 2.99 | [22] |
| C-MXene/C-HNF | 0.5/0.01 M NaCl | 0.03 | | 1.09 | [23] |
| 2D-NNF | 0.5/0.01 M NaCl | 0.03 | | 8.61 | [24] |
| PBONM | 0.5/0.01 M NaCl | 0.03 | | 7.7 | [25] |
| GPPS | 0.5/0.01 M NaCl | 0.0314 | | 8.65 | [26] |
| H-MXene | 0.5/0.01 M NaCl | 0.03 | | 9.47 | [27] |
| MgAl-LDH | 0.5/0.01 M NaCl | 0.03 | | 2.26 | [28] |
| EGO-10 | 0.5/0.01 M NaCl | 0.041 | | 5.32 | [29] |
| 3D SCOF | 0.5/0.01 M NaCl | 0.03 | | 21.2 | [30] |
| TFPT-TMT COF | 0.5/0.01 M NaCl | 0.00785 | | 13.3 | [31] |
| PAA-cPEI | 0.5/0.01 M NaCl | 0.008 | | 3.7 | [32] |
| *p*-BCP-1 | 0.5/0.01 M NaCl | 0.00785 | | 19.3 | [33] |
| MXene fibers | 0.5/0.01 M NaCl | 0.0012 | | 12.3 | [34] |
| ZnTPP-COF | real seawater/river water | 0.006 | | 14.63 | [35] |
| h-PEI capped BCP | 0.5/0.01 M NaCl | 0.008 | | 13.2 | [36] |
| TpPa-(SO_3_H)_1_ COF | 0.5/0.01 M NaCl | 0.0785 | | 24.53 | This work |
| TpPa-(SO_3_H)_1.5_ COF | 0.5/0.01 M NaCl | 0.0785 | | 19.55 |  |

**References**

[1] J. Zhang, H. Zhang, Y. R. Kong, L. Zhou, S. Li, L. Zhuang, N. Li, X. M. Ren, Z. Xu, *J. Am. Chem. Soc.* **2025**, 22, 18934.

[2] L. P. Zeng, Q. He, Y. C. Liao, S. Y. Kuang, J. C. Wang, W. Ding, Q. Liao, Z. D. Wei, *Research* **2020**, *2020*, 4794706.

[3] P. P. Zuo, Y. Y. Li, A. Q. Wang, R. Tan, Y. H. Liu, X. Liang, F. M. Sheng, G. G. Tang, L. Ge, L. Wu, Q. L. Song, N. B. McKeown, Z. J. Yang, T. W. Xu, *Angew. Chem. Int. Ed.* **2020**, *59*, 9564.

[4] Z. Q. Li, J. Guo, J. F. Zheng, T. A. Sherazi, S. H. Li, S. B. Zhang, *Macromolecules* **2020**, *53*, 10998.

[5] W. J. Song, K. Peng, W. Xu, X. Liu, H. Q. Zhang, X. Liang, B. J. Ye, H. J. Zhang, Z. J. Yang, L. Wu, X. L. Ge, T. W. Xu, *Nat. Commun.* **2023**, *14*, 2732.

[6] R. Tan, A. Q. Wang, R. Malpass-Evans, R. Williams, E. W. Zhao, T. Liu, C. C. Ye, X. Q. Zhou, B. P. Darwich, Z. Y. Fan, L. Turcani, E. Jackson, L. J. Chen, S. Y. Chong, T. Li, K. E. Jelfs, A. I. Cooper, N. P. Brandon, C. P. Grey, N. B. McKeown, Q. L. Song, *Nat. Mater.* **2020**, 19, 195.

[7] P. P. Zuo, C. C. Ye, Z. R. Jiao, J. Luo, J. K. Fang, U. S. Schubert, N. B. McKeown, T. L. Liu, Z. J.Yang, T. W. Xu, *Nature* **2023**, 617, 299.

[8] A. Q. Wang, C. Breakwell, F. Foglia, R. Tan, L Lovell, X. C. Wei, T. Wong, N. Q. Meng, H. D. Li, A. Seel, M. Sarter, K. Smith, A. Alvarez-Fernandez, M. Furedi, S. Guldin, M. M. Britton, N. B. McKeown, K. E. Jelfs, Q. L. Song, *Nature* **2024**, 635, 353.

[9] C. C. Ye, A. Q. Wang, C. Breakwell, R. Tan, C. G. Bezzu, E. Hunter-Sellars, D. R. Williams,N. P. Brandon, P. A. A. Klusener, A. R. Kucernak, K. E. Jelfs, N.B. McKeown, Q. L. Song, *Nat. Commun.* **2022**, 13, 3184.

[10] X. D. You, L. Cao, Y. W. Liu, H. Wu, R. L. Li, Q. X. Xiao, J. Q. Yuan, R. N. Zhang, C. Y. Fan, X. Y. Wang, P. F. Yang, X. Y. Yang, Y. Ma, Z. Y. Jiang, *ACS Nano* **2022,** 16, 11781.

[11] H. S. Sasmal, H. B. Aiyappa, S. N. Bhange, S. Karak, A. Halder, S. Kurungot, R. Banerjee, *Angew. Chem. Int. Ed.* **2018**, 57, 10894.

[12] Z. F. Wang, Q. Yu, Y. B. Huang, H. D. An, Y. Zhao, Y. F. Feng, X. Li, X. L. Shi, J. J. Liang, F. S. Pan, P. Cheng, Y. Chen, S. Q. Ma, Z. J. Zhang, *ACS Cent. Sci.* **2019**, 5, 1352.

[13] C. H. Ding, M. Breunig, J. Timm, R. Marschall, J. Senker, S. Agarwal, *Adv. Funct. Mater.* **2021**, 31, 2106507.

[14] X. Y. Wang, B. B. Shi, H. Yang, J. Y. Guan, X. Liang, C. Y. Fan, X. D. You, Y. A. Wang, Z. Zhang, H. Wu, T. Cheng, R. N. Zhang, Z. Y. Jiang, *Nat. Commun*. **2022**, 13, 1020.

[15] X. Ma, N. Sun, Z. G. Li, M. M. Tong, Q. Ding, Z. F. Wang, L. Bai, L. L. Dong, Y. Liu, *Adv. Funct. Mater.* **2024**, 34, 2312203.

[16] X. Y. He, Y. Yang, H. Wu, G. W. He, Z. X. Xu, Y. Kong, L, Cao, B. B. Shi, Z. J. Zhang, C. Tongsh, K, Jiao, K. Y. Zhu, Z. Y. Jiang, *Adv. Mater.* **2020**, 32, 2001284.

[17] Z. Zhang, Y. X. Gong, M. Liu, M. Chen, C. C. Yin, M. J. Wie, Y. Wang, *Angew. Chem. Int. Ed.* **2025**, 64, e18250.

[18] H. Yang, L. Yang, H. Wang, Z. Xu, Y. Zhao, Y. Luo, N. Nasir, Y. Song, H. Wu, F. Pan, Z. Jiang, *Nat. Commun.* **2019**, 10, 2101.

[19] H. Yu, J. Guan, Y. Chen, Y. Sun, S. Zhou, J. Zheng, Q. Zhang, S. Li, S. Zhang, *Small* **2024**, *20*, 2305613.

[20] Y. Kong, B. Lyu, C. Y. Fan, Y. Yang, X. Y. Wang, B. B. Shi, J. W. Jiang, H, Wu, Z. Y. Jiang, *J. Am. Chem. Soc.* **2023**, 145, 27984.

[21] R. L. David, CRC Handbook of Chemistry and Physics, Internet Version 2005, CRC Press, Boca Raton, FL, **2005**.

[22] J. Xiao, M. Cong, M. Li, X. Zhang, Y. Zhang, X. Zhao, W. Lu, Z. Guo, X. Liang, G. Qing, *Adv. Funct. Mater.* **2024**, 34, 2307996.

[23] J. Rao, Z. W. Lv, X. Q. Yan, J. Pan, G. G. Chen, B. Z. Lü, F. Peng, *Adv. Funct. Mater.* **2024**, 34, 2309869.

[24] J. D. Tang, Y. Wang, H. Y. Yang, Q. Q. Zhang, C. Wang, L. Y. Li, Z. L. Zheng, Y. H. Jin, H. Wang, Y. F. Gu, T. Y. Zuo, *Nat. Commun.* **2024**, 15, 3649.

[25] R. Y. Duan, J. L. Zhou, X. Zheng, X. Y. Ma, R. Zhai, J. R. Hao, Y. H. Zhou, C. Teng, L. Jiang, *Adv. Funct. Mater.* **2024**, 34, 2311258.

[26] H. Zhi, P. Yan, D. J. Wang, Y. X. Liu, J. B. Tang, X. Yang, Z. X. Liu, Y. F. Zhang, N. B. Li, M. An, H. Liu, G. B. Xue, *Adv. Funct. Mater.* **2024**, 34, 2401922.

[27] H. Qian, P. G. Peng, H. Z. Fan, Z. Yang, L. X. Yang, Y. G. Zhou, D. Tan, F. Y. Yang, M. Willatzen, G. Amaratunga, Z. L. Wang, D. Wei, *Angew. Chem. Int. Ed.* **2024**, 136, e202414984.

[28] S. Qin, G. L. Yang, S. A. Wang, Y. X. Ma, Z. Y. Wang, L. F. Wang, D. Liu, W. W. Lei, *Small* **2024**, 20, 2400850.

[29] K. R. Bang, C. Kwon, H. Lee, S. Kim, E. S. Cho, *ACS Nano* **2023**, 17, 10000.

[30] T. H. Zhu, Y. Kong, B. Lyu, L. Cao, B. B. Shi, X. Y. Wang, X. Pang, C. Y. Fan, C. Yang, H. Wu, Z. Y. Jiang, *Nat. Commun.* ***2023***, 14, 5926.

[31] K. Wang, H. Y. Yang, Z. Q. Liao, S. X. Li, M. Hambsch, G. E. Fu, S. C. B. Mannsfeld, Q. Sun, T. Zhang, *J. Am. Chem. Soc.* **2023**, 145, 5203.

[32] X. Q. Yang, C. Li, P. X. Liu, Y. Zhai, I. Hussain, X. Sui, L. C. Gao, L. Jiang, *Chem. Mater.* **2023**, 35, 7266.

[33] C. Li, H. M. Jiang, P. X. Liu, Y. Zhai, X. Q. Yang, L. C. Gao, L. Jiang, *J. Am. Chem. Soc.* **2022**, 144, 9472.

[34] F. Hashemifar, A. Esfandiar, *J. Mater. Chem. A* **2022**, 10, 24915.

[35] J. L. Yang, B. Tu, G. J. Zhang, P. C. Liu, K. Hu, J. R. Wang, Z. Yan, Z. W. Huang, M. N. Fang, J. J. Hou, Q. J. Fang, X. H. Qiu, L. S. Li, Z. Y. Tang, *Nat. Nanotechnol.* **2022**, 17, 622.

[36] C. Li, L. P. Wen, X. Sui, Y. R. Cheng, L. C. Gao, L. Jiang, *Sci. Adv.* **2021**, 7, eabg2183.
